# How climate change-related abiotic factors affect the production of industrial valuable compounds in Lamiaceae plant species: a review

**DOI:** 10.3389/fpls.2024.1370810

**Published:** 2024-07-10

**Authors:** Inês Mansinhos, Sandra Gonçalves, Anabela Romano

**Affiliations:** Mediterranean Institute for Agriculture, Environment and Development (MED) and CHANGE – Global Change and Sustainability Institute, Faculdade de Ciências e Tecnologia, Universidade do Algarve, Campus de Gambelas, Faro, Portugal

**Keywords:** bioactivity, climate change, environmental stress, essential oils, phenolic compounds

## Abstract

The interest in medicinal and aromatic plants (MAPs) has increased significantly in recent years, driven by the growing demand for natural products. MAPs are a valuable source of secondary metabolites, which renders them useful to a number of industries, including cosmetics, pharmaceuticals, and food. The Lamiaceae family includes economically important MAPs that produce valuable secondary metabolites such as essential oils (EOs) and phenolic compounds (PCs). The quantity and quality of these secondary metabolites are affected by abiotic stress factors. In a climate change scenario, the Lamiaceae is one of the most affected families, especially due to its wide distribution in the Mediterranean region. In the present study, the most common climate-related environmental stress factors, namely, drought, salinity, temperature, light, and heavy metals, were reviewed and discussed in order to assess their impact on the chemical profiles of EOs and PCs, as well as on the biological properties (antioxidant, antibacterial, antimelanogenic, pest-repellent, and UV-protective) of Lamiaceae species. It can be posited that these stresses typically act as a catalyst for the secondary metabolism of these plants, resulting in increased production of EO compounds (e.g., 1,8-cineole, linalool, camphor, borneol, and limonene) and PCs (e.g., rosmarinic, caffeic, and salvianolic acids) and subsequent enhancement of their biological activities. In view of the industrial applications of these bioactive compounds, it is of interest to explore the changes in secondary metabolism induced by environmental factors as it is possible to increase the accumulation of valuable secondary metabolites.

## 1 Introduction

**Figure 1:**
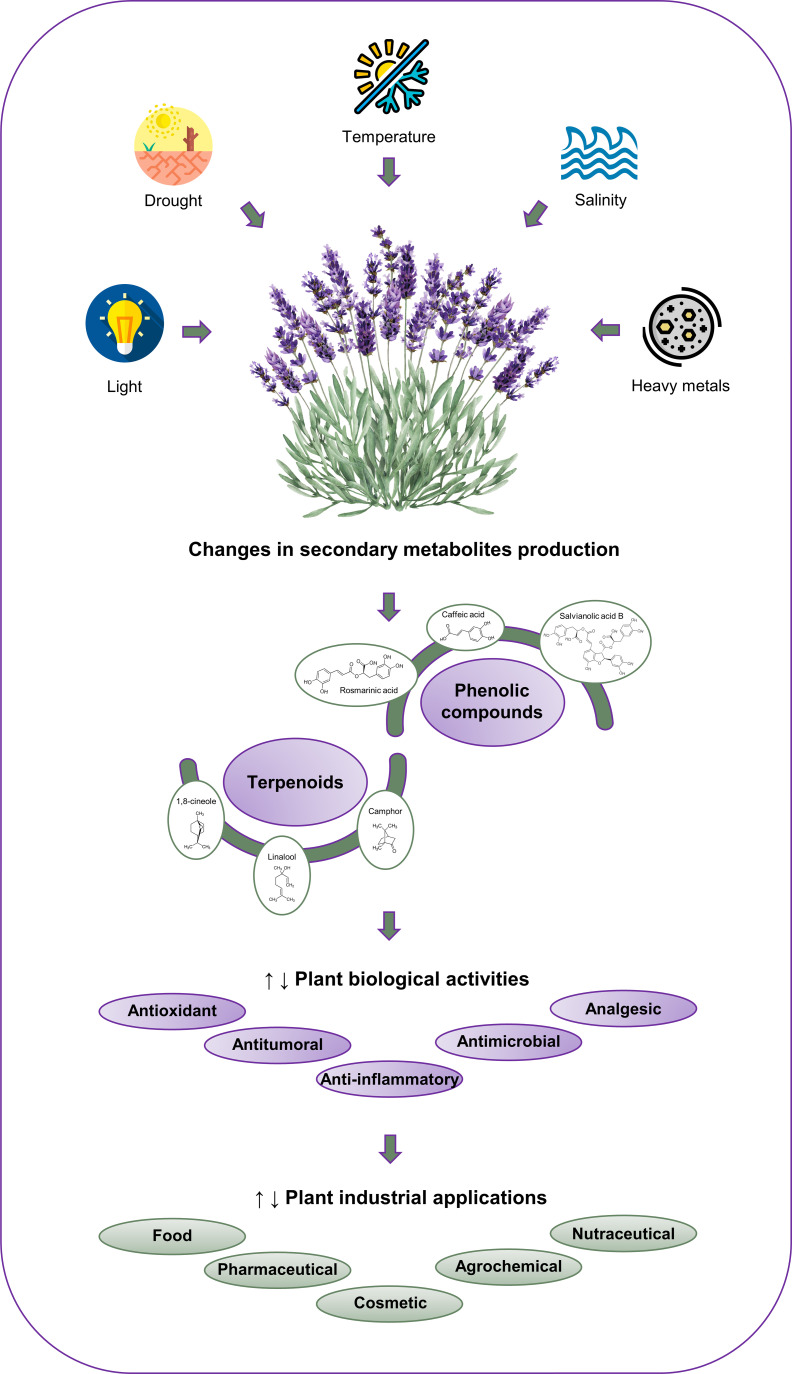
Effect of different abiotic stress factors (drought, salinity, temperature, light and heavy metals) on the production of the main Lamiaceae secondary metabolites and their potential industrial applications.

Globally, plant growth and development are increasingly affected by climate change-related factors. This is mainly due to the fact that these stresses decrease the uptake and diffusion of CO_2_ and modify many biochemical reactions. Indeed, these abiotic stresses represent the primary cause of losses in agricultural productivity, with a reduction in yield of over 50% observed in the agriculture sector (Mahajan et al., 2020). In numerous regions, the most significant and detrimental factors are drought and salinity. It is estimated that over 50% of the arable land will be salinized by 2050 (Jan et al., 2018). Another crucial factor that has been stimulated by anthropogenic global warming is temperature. It is anticipated that the frequency and intensity of heat waves and extremely warm summers will increase, which will result in an exacerbation of drought events (Lehner et al., 2018). An essential physical factor involved in plant growth and development is light/radiation, which is particularly influenced by photoperiod (duration of illumination), quality (wavelength, color), and quantity (fluence rate) (Mahajan et al., 2020). In the context of climate change, ultraviolet (UV) radiation, particularly UV-B radiation, is likely to have a significant impact on plants due to its high capacity to cause biological damage (Mansinhos et al., 2024). Furthermore, climate change also affects the pathways of heavy metal pollution, including photo-enhanced toxicity, uptake, metabolism, biodegradation, adsorption, photodegradation, volatilization, and hydrolysis (Mok et al., 2023). The impact of climate change is of particular concern for endemic and rare species, which are at high risk of extinction (Kougioumoutzis et al., 2022). It is of utmost importance to gain a comprehensive understanding of the responses of plants to these constraints if we are to enhance food security and minimize economic losses (Butcher et al., 2016). The effects of climatic changes result in plants being subjected to conditions that exceed their physiological homeostasis, thereby necessitating adaptation. Plants typically respond to stressful conditions by reducing or inhibiting growth, inhibiting photosynthesis, and activating several tolerance mechanisms, including the accumulation of osmolytes, enzymatic and non-enzymatic antioxidant systems, and the expression of specific defense proteins (Szekely-Varga et al., 2020b). Secondary metabolites, such as phenolics, alkaloids, and terpenoids, play an important role in the adaptation and recovery of plants to environmental conditions, mainly due to their ability to scavenge reactive oxygen species (ROS) (Albergaria et al., 2020; Mahajan et al., 2020). It has been reported that environmental stress conditions affect both the composition and concentration of these metabolites, as well as their biological properties (e.g., antioxidant, antitumor, antimicrobial, anti-inflammatory, gastrointestinal, and cardioprotective) (Albergaria et al., 2020) and the consequent health benefits and/or industrial applicability (e.g., food, pharmaceutical, cosmetic, and agrochemical) (Nabi et al., 2021) (**Figure 1**).

The demand for medicinal and aromatic plants (MAPs) that produce a broad range of secondary metabolites has increased radically in recent years. Geographically, the global herbal medicine market is segmented into the Americas, Europe, Asia-Pacific, the Middle East, and Africa. In 2021, the global market value of MAP products (prepared in the form of extracts, powders, capsules, tablets, and syrups) was approximately US$145 billion and is expected to reach more than US$356 billion by 2030 (Herbal Medicine Market, 2021). In addition, billions of people worldwide depend (partially or completely) on more than 70,000 MAPs. According to the World Health Organization (WHO), it is estimated that 88% of all countries use traditional medicine such as herbal medicines, yoga, acupuncture, indigenous therapies, and others. In terms of pharmaceutical formulations, more than 40% are based on natural products and breakthrough drugs derived from traditional medicine (e.g., aspirin and artemisinin) (WHO, 2022). Unfortunately, the natural stocks of MAPs are declining due to the increasing demand for them, which is closely linked to changes in the external climatic context (Rahman et al., 2022). The Lamiaceae family is of great significance in the realm of MAPs, as it is responsible for the production of essential oils (EOs) and phenolic compounds (PCs) with industrial applications (Mamadalieva et al., 2017). Although there are some review articles reporting the effects of environmental stress factors related to climate change on MAP secondary metabolite production, a systematic review on Lamiaceae plants is lacking. Consequently, the objective of this study is to provide a comprehensive overview of the impact of environmental stress conditions on the production of EOs and PCs in this significant family of plants which is anticipated to be one of the most affected by climate change due to its extensive distribution in the Mediterranean region.

## 2 Lamiaceae family: general description, economic importance, and main secondary metabolites

Lamiaceae is a very important angiosperm family consisting of aromatic, medicinal, and ornamental plants. It is the largest family in the order Lamiales, comprising 236 genera with more than 7,000 species, mostly herbs and shrubs (Assaf et al., 2022; Diab et al., 2022). Among the genera, the largest belonging to this family is *Salvia* (900), followed by *Scutellaria* (360), *Stachys* (300), *Plectranthus* (300), *Hyptis* (280), *Teucrium* (250), *Vitex* (250), *Thymus* (220), and *Nepeta* (200) (Tamokou et al., 2017). This family is distributed throughout the world, with a particular incidence in the Mediterranean and subtropical regions. Morphologically, the stems of these plants are usually square in cross section (with some exceptions), and the flowers are bilaterally symmetrical with five united petals and five united sepals. They are usually bisexual and verticillastrate (a flower cluster that appears to be a whorl of flowers but is actually two crowded clusters) (Raja, 2012). The plants are often aromatic in all parts (Raja, 2012), and for this, the Lamiaceae family has significant economic importance due to their EOs or use as culinary herbs/spices (Khojasteh et al., 2020). For culinary use, oregano and marjoram (*Origanum*), thyme (*Thymus*), sage (*Salvia*), mint (*Mentha*), rosemary (*Rosmarinus*), basil (*Ocimum*), and lavender (*Lavandula*) are the most commonly used species. In addition, several Lamiaceae genera (e.g., *Salvia*, *Lavandula*, *Teucrium*, *Phlomis*) are also cultivated as ornamental garden/houseplants (Patrignani et al., 2021) due to their aromatic properties and ease of cultivation. In fact, these plants are among the easiest plants to propagate by stem cuttings (Raja, 2012). From an industrial and economic perspective, these MAPs are of significant importance to the development of the cosmetics, agro-alimentary, pharmaceutical, and perfume sectors (Avasiloaiei et al., 2023). *Mentha* and *Lavandula* are two important genera that are widely used in cosmetics. Species such as *Rosmarinus officinalis* L., *Origanum majorana* L., and *Ocimum basilicum* L. are commonly used as flavorings in the food industry and *Satureja montana* L., *Mentha* x *piperita* L., *Salvia officinalis* L., and *Sideritis scardica* Griseb. as beverages and teas (Patrignani et al., 2021). All these species are known to be rich sources of bioactive compounds, especially PCs and terpenes.

Lamiaceae plants have a long traditional history based on their medicinal use (Patrignani et al., 2021) as anti-inflammatory, sedative, and analgesic agents. In addition, these plants have also shown great applicability as common teas, flavors, and insect repellents. Most of the species belonging to this family are aromatic and contain a mixture of bioactive compounds used in many industries (Mamadalieva et al., 2017), mainly in the form of EOs (Chakrabartty et al., 2022). In addition to EOs, these plants can be used as extracts or isolated bioactive compounds (Mamadalieva et al., 2017; Patrignani et al., 2021). Plants produce different types of bioactive compounds, which are classified into three main groups based on their origin: terpenoids, PCs, and nitrogen-containing compounds (Mahajan et al., 2020). Lamiaceae plants mainly produce terpenes (terpenoids) and PCs (Mamadalieva et al., 2017).

EOs are volatile and highly concentrated hydrophobic liquids isolated by distillation from different parts of plants, specifically flowers, leaves, fruits, stems, bark, peels, roots, seeds, or whole plants from a single botanical source (Gonçalves et al., 2020; Masyita et al., 2022). The chemical constituents of EOs are classified into terpenes, terpenoids, phenylpropanoids, and other components (Masyita et al., 2022). While terpenes are simple hydrocarbons containing isoprene units (five carbons), terpenoids are more complex terpenes with additional functional groups. Terpenoids (oxygenated hydrocarbons) contain oxygen molecules that are formed through biochemical variations by the removal or addition of methyl groups. However, both terms (terpenes and terpenoids) are often used interchangeably (Nagegowda and Gupta, 2020; Masyita et al., 2022). Plants produce two main classes of terpenoids: the primary (essential) terpenoid metabolites (such as carotenoids, sterols, and many hormones) and the secondary (non-essential) or specialized terpenoid metabolites (terpenes). The most abundant group of terpenoids—terpenes—are often specific or unique to individual plant species or groups of species and provide the plant with overall fitness to interact with the environment (Nagegowda and Gupta, 2020). Depending on the number of isoprene units, terpenes are classified into different categories, namely, hemiterpenes formed by one isoprene unit (C5), monoterpenes (C10), sesquiterpenes (C15), diterpenes (C20), triterpenes (C30), and tetraterpenes (C40). Monoterpenes are the major compounds of EOs (90%), followed by sesquiterpenes (Masyita et al., 2022). In plants, terpene biosynthesis occurs via the mevalonate (MVA) pathway (localized in the cytosol) and the 2-methylerythritol 4-phosphate (MEP) pathway (localized in the plastid) and involves different enzymatic reactions (Senji and Mandoulakani, 2018; Gonçalves et al., 2020). Monoterpenes, diterpenes, and tetraterpenes are synthesized in the plastid, whereas sesquiterpenes and triterpenes are synthesized in the cytosol (Mahajan et al., 2020). EOs and their individual compounds have a wide range of biological activities (e.g., antimicrobial, anti-inflammatory, analgesic, antioxidant), and more than 3,000 EOs have shown industrial importance, especially in perfumery and cosmetics (Senji and Mandoulakani, 2018; Gonçalves et al., 2020). The emission of these bioactive compounds depends on several environmental factors (Mahajan et al., 2020).

PCs are an important group of secondary metabolites produced in response to abiotic stress. Chemically, they are defined as compounds containing more than one hydroxyl group that is glycosylated or methylated (Mahajan et al., 2020). These compounds are divided into five major subclasses: phenolic acids, flavonoids, stilbenes, tannins, and lignans. In terms of secondary metabolism pathways, the shikimate and malonate pathways are responsible for the biosynthesis of the precursors of phenolics (Gonçalves et al., 2020). In plants, the shikimate pathway is responsible for most PC biosynthesis, whereas in bacteria and fungi, the malonate pathway is more important for their biosynthesis (Mahajan et al., 2020). As they are derived from phenylalanine, PCs are also known as phenylpropanoids (Sharma et al., 2019). Due to the presence of hydroxyl and carboxyl functional groups, phenolics have the ability to chelate heavy metal ions, such as iron, manganese, and copper, and to scavenge free radicals. In addition to their antioxidant capacity, other pharmacological properties (e.g., antitumor, antimicrobial, anti-inflammatory, gastrointestinal, and cardioprotective) have been widely attributed to different phenolic species (Albergaria et al., 2020).

## 3 Methodology

In light of the considerable medicinal, industrial, and economic value of Lamiaceae species, a review was conducted of the literature published over the past two decades (2005–2024) on the impact of environmental stress conditions, namely, drought, salinity, temperature, light, and heavy metals, on the production of secondary metabolites (EOs and PCs) and the biological properties of these species. A comprehensive search was conducted across a range of scientific online databases, including Web of Science, PubMed, Scopus, Science Direct, and Google Scholar. The following search terms were used: “Lamiaceae,” “environmental stress,” “abiotic stress,” “drought,” “water stress,” “salinity,” “salt stress,” “temperature,” “heat stress,” “cold stress,” “light stress,” “UV-B stress,” “heavy metals,” “plant secondary metabolites,” “bioactive compounds,” “essential oils,” “phenolic compounds,” and “biological activities.” This review has assembled a total of 180 references, comprising research and review articles, book chapters, a case study, and some clinical trial reports. The present review article presents five tables, separated into EOs and PCs, which indicate mostly the individual compounds that are affected by the abiotic stresses previously mentioned. Furthermore, a figure has been included to illustrate the impact of abiotic stress factors on the production of the main PCs and terpenoids from Lamiaceae MAPs, as well as their biological properties and potential industrial applications.

## 4 Effect of environmental stress factors on secondary metabolites production by Lamiaceae species

Climate change is expected to have a major impact on Lamiaceae species due to their wide distribution in the Mediterranean basin. This region is characterized by a large number of endemic plants and enormous diversity and is even considered the second largest terrestrial biodiversity hotspot in the world (Tomou et al., 2022). According to recent data, the number of days above 37°C is expected to double from 30 to 60 by 2050 in northern Africa, southern Spain, and Turkey. These rising temperatures will increase the risk of droughts, water stress in basins, wildfires, and floods and significantly alter the Mediterranean climate. Spain, Italy, Portugal, and parts of Turkey and Greece are expected to experience at least 6 months of drought each year by 2050. Basin water supplies could decline by approximately 10% by 2030 and up to 25% by 2050 (McKinsey Global Institute, 2020). These environmental changes could affect the distribution of these MAPs, causing them to move to more climatically appropriate and suitable habitats. This could lead to financial problems if economically important plants of local rural communities are lost from locally accessible areas (Kougioumoutzis et al., 2022). In addition, the significant decline in the populations of some MAPs is mainly due to anthropogenic activities (e.g., uncontrolled collection, expansion of the urban/tourist areas, deforestation, fires). Oregano, thyme, and sage are three examples of Lamiaceae plants that have a very limited distribution in some areas and that are threatened with extinction mainly due to uncontrolled collection by free gatherers (Tomou et al., 2022). Thus, given the environmental and human causes, the conservation of these species must be of public interest to protect biodiversity and its sustainable management.

The industrial applicability of Lamiaceae plants is related to their biological properties, which are known to be dependent on their secondary metabolites. However, the production of these compounds is sensitive to small variations in the surrounding environment (Assaf et al., 2022). Drought, salinity, temperature, light and heavy metals are the main stresses that plants face today (Mahajan et al., 2020). The following is an update of studies from the last two decades on the secondary metabolic responses of Lamiaceae species to the five abovementioned abiotic stresses.

### 4.1 Drought

Water scarcity, one of the most damaging stresses, is responsible for the productivity losses in several crops and MAPs, especially in arid and semiarid regions (Albergaria et al., 2020). This abiotic stress, which is expected to increase in duration, frequency, and intensity as a result of climate change, affects the physiological and biochemical processes of several plants (Moustakas et al., 2022). Under water limitation, roots send a chemical signal through the xylem to the leaves, stimulating a partial stomatal closure to reduce water loss (transpiration) and prevent dehydration. As a result, there is a direct decrease in the CO_2_ uptake (which is detrimental to photosynthesis) and, since NADPH is not used in the Calvin cycle, NADP^+^ is not available. These changes lead to increased levels of free radicals and ROS (Albergaria et al., 2020; Karalija et al., 2022; Moustakas et al., 2022), which negatively affect a wide range of plant processes, such as growth and development, loss of turgor leading to cell dehydration, photosynthesis, and many other metabolic parameters (Jan et al., 2018; Khare et al., 2020; Moustakas et al., 2022). Although some researchers suggest a positive effect of elevated CO_2_ (resulting from fossil fuels burned by humans for energy and exacerbating the greenhouse effect) on crop yield by increasing photosynthesis, this does not appear to be sufficient to counteract the negative effects of severe drought on photosynthesis and yield (Moustakas et al., 2022). Drought tolerance traits are correlated with the accumulation of several compounds. Plants accumulate various organic solutes known as osmoregulators, osmoprotectants, or osmolytes (e.g., proline, soluble sugars, glycine betaine) to help cells maintain turgor pressure in their cytosol. Furthermore, another strategy to cope with drought stress is the enzymatic [e.g., catalase (CAT), superoxide dismutase (SOD), glutathione reductase (GR), ascorbate peroxidase (APX)] and non-enzymatic antioxidant defense systems (e.g., glutathione, ascorbate, α-tocopherol, carotenoids, PC) capable of reducing ROS (Khare et al., 2020; Karalija et al., 2022; Moustakas et al., 2022).

**Table 1** summarizes the effect of drought on EO and PC production in several Lamiaceae species. *Salvia* spp. are among the most important MAPs, and several studies have been conducted to evaluate their ability to respond to drought stress. Ramezani et al. (2020) investigated the effect of water deficit on the EO composition, namely, the five main metabolites (1,8-cineole, camphor, borneol, α- and β-thujone), in three genotypes of *Salvia* spp. [*S. officinalis*, *S. reuterana* (Yasuj), and *S. reuterana* (Urmia)] and showed that genetic differences could influence the quality and quantity of these compounds. In *Salvia dolomitica* Codd, drought stimulated the biosynthesis of sesquiterpene hydrocarbons, an important class of terpenoids for the pharmaceutical, food, and cosmetics industries. However, a negative effect was observed for monoterpene hydrocarbons (Caser et al., 2019). Camphor, the main oxygenated monoterpene in *Salvia sinaloensis* Fern (Caser et al., 2018) and *Salvia fruticosa* Mill (Chrysargyris et al., 2016) increased after water stress conditions. Similarly, in *S. officinalis*, the amount of monoterpenes increased tremendously in response to water stress, doubling after only 2 days (Radwan et al., 2017). In *S. officinalis*, all the EO subclasses were increased after drought exposure (Bettaieb et al., 2009).

**Table 1:** Alteration in secondary metabolite (essential oils and phenolic compounds) production in Lamiaceae species under drought stress.

Plant species	Reported effect	Reference
Essential oils		
*Dracocephalum moldavica* L.	↑ EO content	Kamalizadeh et al. (2019)
*Lavandula angustifolia* Mill.	↑ 1,8-Cineole, borneol ↓ α-Pinene, sabinene, β-pinene, D-limonene	Chrysargyris et al. (2016)
	↑ α-Pinene, β-pinene, β-phellandrene, 3-carene, endo-borneol ↓ 1,8-Cineole, camphor	Kulak (2020)
*Lavandula latifolia* Med. It	↓ EO content	García-Caparrós et al. (2019)
*Melissa officinalis L.*	↑ EO content	Szabó et al. (2017)
*Ocimum basilicum* L.	↑ EO yield	Pirbalouti et al. (2017)
	↑ α-Pinene, 1,8-cineole, D-limonene, methyl chavicol, bergamotone ↓ Linalool, methyleugenol	Kulak (2020)
*Ocimum tenuiflorum* L.	↑ Eugenol, methyleugenol, β-caryophyllene	Nguyen et al. (2022)
*Origanum vulgare* subsp*. gracile*	↑ EO content ↓ ρ-Cymene	Morshedloo et al. (2017)
*Origanum vulgare* subsp*. virens*	↑ (E)-β-Caryophyllene, (Z)-b-ocimene, β-bisabolene ↓ Sabinene	Morshedloo et al. (2017)
*Rosmarinus officinalis* L.	↑ Camphor, linalool, 1,3-cyclopentadiene, 1,2,5,5-tetramethyl, D-verbenone, limonene, cyclohexane ↓ Bornyl acetate, caryophyllene oxide, β-pinene, *trans*-verbenol, β-myrcene, linalyl isobutyrate, benzenemethanol, α-methyl dibutylphtalate	Sarmoum et al. (2019)
	↑ Camphene, D-limonene, camphor ↓ α-Pinene, 1,8-cineole	Kulak (2020)
*Salvia dolomitica* Codd	↑ β-Caryophyllene, germacrene D, δ-cadinene, bicyclogermacrene, α-guaiene, α-β-humulene, borneol, Δ-elemene, isoledene, α-copaene, β-cubebene, β-elemene ↓ Limonene, Δ-3-carene, (E)-β-ocimene, α-pinene, camphene, β-pinene, myrcene	Caser et al. (2019)
*Salvia fruticose* Mill.	↑ Camphor, α-thujone ↓ α-Pinene, camphene, β-myrcene, D-limonene, 1,8-cineole, terpinolene	Chrysargyris et al. (2016)
*Salvia officinalis* L.	↑ Camphor, 1,8-cineole ↓ α-Pinene, camphene, α-thujone, β-thujone	Kulak (2020)
	↑ Borneol, camphor, 1,8-cineole ↓ α-Thujone, β-thujone	Ramezani et al. (2020)
	↑ Camphor, 1,8-cineole, α-thujone, β-thujone	Radwan et al. (2017)
	↑ Camphor, β-thujone, 1–8-cineole, terpinene-4-ol, linalool, α-terpineol, bornyl acetate, viridiflorol, manool ↓ α-Humulene, α-thujone	Bettaieb et al. (2011)
*Salvia sclarea* L.	↓ EO content	García-Caparrós et al. (2019)
*Salvia reuterana* (Yasuj)	↑ Borneol ↓ α-Thujone, 1,8-cineole	Ramezani et al. (2020)
*Salvia reuterana* (Urmia)	↓ Borneol, α-thujone, 1,8-cineole	Ramezani et al. (2020)
*Salvia sinaloensis* Fern.	↑ Camphor, camphene ↓ Germacrene D	Caser et al. (2018)
*Sideritis perfoliata* L. subsp*. perfoliata*	↑ EO yield	Chrysargyris et al. (2019a)
*Thymus daenensis* Celak	↓ EO content	Arpanahi et al. (2020)
	↑ Carvacrol, β-myrcene, 1,8-cineole ↓ Thymol	Pirbalouti et al. (2014)
*Thymus vulgaris* L.	↑ γ-Terpinene, α-thujone, camphene, ρ-cymene, borneol ↓ Thymol, carvacrol, α-pinene, β-caryophyllene	Razavizadeh et al. (2019)
	↓ EO content	Arpanahi et al. (2020)
	↑ Thymol, borneol ↓ ρ-Cymene, α-pinene, γ-terpinene, linalool, β-caryophyllene	Khalil et al. (2018)
	↑ ρ-Cymene, linalool, carvacrol ↓ α-Terpinene, α-terpineol, thymol methyl ether	Mohammadi et al. (2018)
	↑ EO content; thymol, α-thujene, β-caryophyllene ↓ EO yield	Said-Al Ahl et al. (2019)
	↑ 1,8-Cineole	Llorens-Molina and Vacas (2017)
	↑ Carvacrol, γ-terpinene, ρ-cymene ↓ Thymol	Alavi-Samani et al. (2015)
Phenolic compounds		
*Dracocephalum moldavica* L.	↑ Rosmarinic acid, chlorogenic acid, ρ-coumaric acid, caffeic acid, apigenin	Kamalizadeh et al. (2019)
*Lavandula angustifolia* Mill.	↑ Total phenolic and flavonoid contents	Szekely-Varga et al. (2020a)
*Lavandula officinalis* L.	↑ Total phenolic content	Mohammadzadeh and Pirzad (2021)
*Melissa officinalis* L.	↑ Total phenolic and flavonoid contents	Szabó et al. (2017)
	↑ Total phenolic and flavonoid contents	Al-Huqail et al. (2020)
	↑ Rosmarinic acid, ferulic acid, caffeic acid, 2,3-dihydroxybenzoic acid, 3,4-dihydroxybenzoic acid, rutin, ρ-coumaric acid	Moshrefi-Araghi et al. (2023)
*Ocimum basilicum* L.	↑ Total phenolic content	Pirbalouti et al. (2017)
*Perovskia atriplicifolia* Benth.	↑ Total phenolic and flavonoid contents	Afshari et al. (2022)
*Perovskia abrotanoides* Karel.	↑ Total phenolic and flavonoid contents	Afshari et al. (2022)
*Rosmarinus officinalis* L.	↑ Total phenolic content	Mohammadzadeh and Pirzad (2021)
*Salvia dolomitica* Codd	↓ Total phenolic and flavonoid contents	Caser et al. (2019)
*Salvia fruticose* Mill.	↑ Total phenolic and flavonoid contents	Chrysargyris et al. (2016)
*Salvia leriifolia* Benth.	↑ Rosmarinic acid, caffeic acid, salvianolic acid B	Hosseini et al. (2020)
*Salvia miltiorrhiza* Bunge	↑ Salvianolic acid B ↓ Rosmarinic acid	Liu et al. (2011)
*Salvia officinalis* L.	↑ Rosmarinic acid, dihydroxybenzoic acid, campherol, gallic acid, *trans*-2-hydroxycinamic acid, chlorogenic acid, ferulic acid, flavone ↓ Cinnamic acid, quercetin 3-D-galactoside, naringin	Bettaieb et al. (2009)
*Scutellaria baicalensis* Georgi	↑ Baicalin	Cheng et al. (2018)
*Sideritis perfoliata* L. subsp*. perfoliata*	↑ Total phenolic and flavonoid contents	Chrysargyris et al. (2019a)
*Thymus lotocephalus* López and Morales	↓ Rosmarinic acid, salvianolic acid A, methylrosmarinic acid, sagerinic acid, luteolin-7-O-glucuronide	Mansinhos et al. (2022a)
*Thymus vulgaris* L.	↑ Total phenolic content	Razavizadeh et al. (2019)
	↑ Total phenolic and flavonoid contents	Khalil et al. (2018)

↑, increase; ↓, decrease.

*Thymus* is another genus that has been well studied in terms of drought tolerance, particularly *Thymus vulgaris* L., a MAP native to the western Mediterranean region of Europe. In soil-grown plants of *T. vulgaris*, water shortage led to a decrease in γ-terpinene content and a significant increase in thymol (Khalil et al., 2018). According to the study by Arpanahi et al. (2020), when *Thymus daenensis* Celak and *T. vulgaris* were subjected to low, mild, and severe water stress, EO content decreased in both species, although this decrease was more pronounced in *T. daenensis*. In other studies, the EO yield and composition in plants of this species differed among plant organs, as well as with developmental periods and growth conditions (Alavi-Samani et al., 2015; Llorens-Molina and Vacas, 2017; Mohammadi et al., 2018). With regard to *in vitro* culture, the major compounds of EOs from *T. vulgaris* callus were differentially affected by drought, with γ-terpinene increasing its content and thymol and carvacrol decreasing (Razavizadeh et al., 2019). The percentage of some chemical compounds in the EOs extracted from *T. daenensis* plants under reduced irrigation was higher than from unstressed plants. Nevertheless, the content of its main component (thymol) was significantly reduced under stressed conditions (Pirbalouti et al., 2014).

To investigate the effect of water shortage on the EO content of five *Melissa officinalis* L. genotypes, plants were irrigated with 40% of the soil water capacity for 3 months, and three of the five cultivars (‘Lorelei,’ ‘Gold Leaf,’ and ‘Quedlinburger Niederliegende’) showed no significant differences between stressed plants and the control. Nevertheless, under drought conditions, the cultivar ‘Soroksar’ increased EO production by 58%, while ‘Lemona’ accumulated only 35% (Szabó et al., 2017). To induce EO biosynthesis, holy basil (*Ocimum tenuiflorum* L. or *O. sanctum* L.) at juvenile and preflowering stages was exposed to drought stress for 5 days. In both stages, the content of three major compounds (eugenol, methyl eugenol, and β-caryophyllene) was significantly enhanced compared to the control (Nguyen et al., 2022).

Six species of the Lamiaceae family commonly cultivated in the Mediterranean region, namely, *M. piperita* (peppermint), *Salvia lavandulifolia* Vahl. (Spanish sage), *Salvia sclarea* L. (clary sage or clear eye), *Thymus mastichina* L. (Spanish marjoram), *Thymus capitatus* (L.) Hoff. et Link., and *Lavandula latifolia* Med., were subjected to water stress, and the EO content was evaluated. The results indicated that the only species affected by drought were *S. sclarea* and *L. latifolia*, with a corresponding decrease in their EO contents (García-Caparrós et al., 2019). Drought affects the chemical profile of EOs from *R. officinalis.* Gas chromatography/mass spectrometry (GC/MS) analysis showed that 10 volatile compounds were identified in the EOs of well-irrigated plants, while 13 volatile compounds were detected in non-irrigated plants. Linalool, limonene, 1,3-cyclopentadiene, 1,2,5,5-tetramethyl, and cyclohexane compounds were characteristic of the plants exposed to drought (Sarmoum et al., 2019). Two different subspecies of *Origanum vulgare* L., namely, ‘*gracile’* and ‘*virens*,’ were exposed to mild and moderate drought, and it was observed that this stress significantly improved the EO content of *gracile* subsp. but did not affect the EO content of *virens* subsp (Morshedloo et al., 2017). The content of oxygenated monoterpenes, especially the major compound 1,8-cineole, was increased in drought-stressed *Lavandula angustifolia* Mill (Chrysargyris et al., 2016), *S. officinalis* (Kulak, 2020), and *O. basilicum* (Kulak, 2020). The yield and content of EOs extracted from plants such as *O. basilicum* (Pirbalouti et al., 2017; Kulak, 2020), *Dracocephalum moldavica* L (Kamalizadeh et al., 2019), *Sideritis perfoliata* L. subsp*. perfoliate* (Chrysargyris et al., 2019a), and *S. officinalis* (Kulak, 2020) were also higher under stressed than unstressed conditions.

As with EOs, several studies have shown that water stress affects PC production in Lamiaceae plants. The content of the main active constituents of *Salvia miltiorrhiza* Bunge (salvianolic acid B) (Liu et al., 2011) and *S. officinalis* (rosmarinic acid) grown in soil (Bettaieb et al., 2011) increased under moderate and severe water deficit. On the other hand, *S. dolomitica* (Caser et al., 2019) and *S. sinaloensis* (Caser et al., 2018) were subjected to moderate and severe drought stress, and total phenolic and flavonoid contents significantly decreased in both species compared to the control. Plantlets and callus cultures of *Salvia leriifolia* Benth. were exposed to polyethylene glycol (PEG) to induce osmotic stress in the culture media, and the levels of rosmarinic acid, caffeic acid, and salvianolic acid B increased significantly in all cultures grown under drought conditions (Hosseini et al., 2020). *Scutellaria baicalensis* Georgi is a traditional MAP known for its high flavonoid content, of which baicalin is the most important. Cheng et al. (2018) showed a significant improvement in baicalin production after subjecting *S. baicalensis* to moderate water stress, but this production decreased under severe stress. Drought stress also favored the production of secondary metabolites in Asiatic Moldavian dragonhead (*D. moldavica*), especially the phenolics rosmarinic, chlorogenic, and ρ-coumaric acids under moderate stress and caffeic acid and apigenin under higher stress conditions (Kamalizadeh et al., 2019). *Thymus vulgaris* plants irrigated with reduced amounts of water achieved an increase in polyphenol and flavonoid contents (Khalil et al., 2018). *Thymus vulgaris in vitro* cultures exposed to PEG-induced osmotic stress obtained the highest amount of total phenolics at 6% PEG (Razavizadeh et al., 2019), while in *Thymus lotocephalus* López and Morales, the total phenolic concentration decreased under 2%, 5%, and 7% PEG (Mansinhos et al., 2022a). Sweet basil (*O. basilicum*) showed an increase in total phenolic (Pirbalouti et al., 2017; Al-Huqail et al., 2020) and flavonoid contents in response to water deficit and an extremely significant decrease in carotenoid content (Al-Huqail et al., 2020). More recently, drought has been shown to improve the contents of the most abundant compounds in *M. longifolia* L., namely, rosmarinic, ferulic, caffeic, 2,3-dihydroxybenzoic, and 3,4-dihydroxybenzoic acids, as well as rutin. Furthermore, drought has been demonstrated to trigger the production of ρ-coumaric acid which was not detected in some accessions under control conditions (Moshrefi-Araghi et al., 2023). Other Lamiaceae species such as *M. officinalis* (Szabó et al., 2017), *Perovskia atriplicifolia* Benth., *Perovskia abrotanoides* Karel (Afshari et al., 2022), and *L. angustifolia* (Szekely-Varga et al., 2020a) improved their phenolic content when subjected to water stress.

### 4.2 Salinity

Soil salinity is known as the accumulation of excess ions, such as chloride (Cl^−^), sodium (Na^+^), sulfate (SO_4_^2−^), magnesium (Mg^2+^), and calcium (Ca^2+^) in the soil (Butcher et al., 2016). This problem has a major impact on soil properties, altering texture and reducing aeration, porosity, and water conductance (Mahajan et al., 2020). According to Butcher et al. (2016), there are two main causes of salinization—primary (natural) and secondary (anthropogenic) salinization. Primary (or dryland), which usually occurs in arid and semiarid regions (with low rainfall and high evapotranspiration), is caused by the capillary rise of saline groundwater and/or by the natural accumulation of salts from a saline parent material. Secondary (or irrigation) salinity has an anthropogenic cause and results from the use of poor-quality irrigation water. The main consequence is a significant inhibition of plant growth, development, and function, as salinity interferes with the uptake of water and nutrients, such as carbon and nitrogen (Cordovilla et al., 2014). This inhibition occurs through two main mechanisms: osmotic/drought stress and specific ionic effects. The first one is related to the change in the osmotic potential of the soil solution surrounding the root, which inhibits the root water uptake. As a result, similar to drought, plants use osmolytes to increase their water uptake. Specific ion effects are physiological effects of individual salt components that accumulate in organic tissues and cause toxicity. A common indicator of most excessive ion levels is membrane damage (Butcher et al., 2016). Another adaptive strategy of plants against stress is the accumulation of secondary metabolites. Several reports have shown that these compounds are significantly affected by salinity and tend to be more abundant in plants exposed to salt stress (Assaf et al., 2022).

Changes in the production of EOs and PCs in Lamiaceae species in response to salinity have been reported by several authors (**Table 2**). *Origanum* is an important genus of the Lamiaceae family. The response of *O. vulgare* plants to soil moisture regimes using fresh and saline water irrigation was evaluated, and it was found that the EO yield decreased significantly with increasing salinity. The amount of carvacrol, the dominant compound in this species (46.44%–77.96%), decreased with salt stress, in contrast to ρ-cymene and γ-terpinene, the second and the third most abundant compounds, respectively (Said-Al Ahl and Wahby, 2010). *Origanum majorana* (syn. *Majorana hortensis* Moench), known as marjoram or sweet marjoram, is a perennial herbaceous plant native to Cyprus and the eastern Mediterranean. The salinity effect is highly significant for the EO content of this species but depends on the severity of the treatment. EO yield increased significantly up to 55.5% at moderate salinity and decreased at high salinity (Jelali et al., 2011). In another study, 50–100 mM of NaCl decreased the EO yield and the content of several individual compounds of marjoram, including the most abundant (*trans*-sabinene hydrate), but increased the percentages of the other predominant compounds, namely, terpinene-4-ol and *cis*-sabinene hydrate (Baâtour et al., 2009). Two years later, the same authors showed that the influence of this stress on the EO composition of marjoram depends on several factors, such as the plant variety (i.e., Tunisian vs. Canadian) (Baâtour et al., 2011). In 19 accessions of *O. basilicum*, EO content was higher under salt stress than under control conditions. All accessions showed a high content of methyl chavicol, an active ingredient of various natural pesticides, pharmaceuticals, and fragrances, and the production of anethole and related structural analogs (Akbari et al., 2018). An increase in EO yield and compound content due to salinity has also been reported in *O. basilicum* plants (Bernstein et al., 2010; Hassanpouraghdam et al., 2011; Tarchoune et al., 2013; Elhindi et al., 2016; Farsaraei et al., 2020). In contrast, different authors showed a negative effect of salt stress on the EO amount of *O. basilicum* major compounds (Bahcesular et al., 2020; Masoudniaragh et al., 2021). More recently, Es-sbihi et al. (2021) investigated how salt stress affected the chemical profile of *S. officinalis* and found important changes in the composition of EO. NaCl decreased the EO yield from 1.2% to 0.4%, and while α-thujone, camphor, and 1,8-cineole were the major compounds of EOs under control treatment, these three components decreased in stressed plants, and thujanone emerged as a new major compound. Other reports also showed a strong effect of salt stress on the EO composition of *Salvia* spp. but with different results, especially depending on the salt concentration (Taarit et al., 2009; Valifard et al., 2014; Kulak et al., 2020). In *S. sclarea*, the application of 25 mM NaCl significantly increased the oil yield which decreased with increasing salt concentration (Taarit et al., 2011). Years later, the same authors found that the transcript of the cineole synthase 1 gene (*SmCin1*) accumulated in the leaves (11-fold) during the first hours of salinity treatment compared to the control.

**Table 2:** Alteration in secondary metabolite (essential oils and phenolic compounds) production in Lamiaceae species under salinity stress.

Plant species	Reported effect	Reference
Essential oils		
*Lavandula angustifolia* Mill.	↓ EO content	Cordovilla et al. (2014)
*Lavandula stoechas* L.	↓ EO content	Valizadeh-Kamran et al. (2019)
	↓ EO content	Vojodi Mehrabani et al. (2017)
*Melissa officinalis* L.	↑ Neryl-acetate, geranyl-acetate, geraniol ↓ α-Citral	Bonacina et al. (2017)
*Mentha* x *piperita* L.	↓ EO content	Khalvandi et al. (2019)
	↑ Menthone, -(−)menthol, myrcene ↓ EO yield, sabinene, linalool, 1,8-cineole, α-terpinolene, isomenthone, isomenthyl acetate	Aziz et al. (2008)
	↓ EO content	Çoban and Göktürk Baydar (2016)
*Mentha pulegium* L.	↑ Limonene, L-linalool, isomenthone, ρ-menthone, p-menth-1-en-8-ol, piperitone, iopiperitenone, piperitenone, -(−)-caryophyllene oxide, humulene oxide ↓ EO yield, pulegone, 3-octanol	Aziz et al. (2008)
*Mentha spicata* L.	↑ Limonene ↓ Carvone	Chrysargyris et al. (2019b)
	↑ Limonene; β-ocimene, myrtenal, *cis*-dihydro carvone, *cis*-carveol, pulegone, β-elemene ↓ Carvone	Chrysargyris et al. (2019c)
*Mentha suaveolens* Ehrh.	↑ Sabinene, myrcene, *trans*-ocimene, L-menthone, 4-terpineol, linalyl propionate, p-menth-1-en-8-ol, nerol, geranyl acetate, germacrene D ↓ EO yield, linalool, neryl acetate	Aziz et al. (2008)
*Ocimum basilicum* cv. Keshkeni luvelu	↑ EO content, sesquiterpene hydrocarbons, oxygenated monoterpenes ↓ Monoterpene hydrocarbons	Farsaraei et al. (2020)
*Ocimum basilicum* L. cv. Genovese	↑ EO yield, methyl eugenol, *trans*-α-bergamotene, α-humulene, spathulenol ↓ (*E*)-β-ocimene, bicyclogermacrene	Tarchoune et al. (2013)
*Ocimum basilicum* L.	↑ EO yield	Akbari et al. (2018)
	↑ Methyl eugenol ↓ Eugenol, linalool, 1–8-cineole, viridiflorene, α-terpineol	Bahcesular et al. (2020)
	↑ EO content	Bernstein et al. (2010)
	↑ EO content; linalool, myrcene, geraniol methylchavicol, farnesol ↓ Eugenol, methyl eugenol, 1,8-cineole	Elhindi et al. (2016)
	↑ Methyl chavicol, borneol, germacrene D, α-amorphene, ∆-cadinene ↓ Linalool, α-(*Z*)-ergamotene, α-cadinol	Hassanpouraghdam et al. (2011)
	↑ Methyl chavicol, 1,8-cineole ↓ α-Bergamotene, methyl eugenol, germacrene D, β-caryophyllene, α-bergamotene	Masoudniaragh et al. (2021)
*Origanum majorana* L.	↑ EO yield, *trans*-sabinene hydrate, linalool, γ-terpinene, terpinolene, β-elemene, geranyl acetate, nerol ↓ Terpinene-4-ol, sabinene, limonene, *cis*-sabinene hydrate, linalyl acetate, bornyl acetate, carvone, β-caryophyllene, bicyclogermacreme, neryl acetate	Baâtour et al. (2009)
	↑ *trans*-Hydrate sabinene, terpinen-4-ol, bicyclogermacreme, geranyl acetate, carvone, linalool, tridecane ↓ *trans*-Hydrate sabinene, sabinene, α-phellandrene, carvone, *cis*-sabinene hydrate	Baâtour et al. (2011)
	↑ Sabinene, *cis*-sabinene-hydrate, terpinen-4-ol, α-terpineol ↓ α-Terpinene, γ-terpinene, *trans-*sabinene-hydrate, α-terpinolene	Jelali et al. (2011)
*Origanum vulgare* L.	↑ Carvacrol ↓ ρ-Cymene	Said-Al Ahl and Wahby (2010)
*Rosmarinus officinalis* L.	↑ EO yield, phellandrene ↓ Dill ether	Dehghani Bidgoli et al. (2019)
	↓ EO yield	Sarmoum et al. (2019)
	↑ Linalool, camphor, borneol, verbenone ↓ α-Pinene, β-pinene, 1,8-cineole	El-Esawi et al. (2017)
	↑ Borneol ↓ 1,8-Cineole	Tounekti et al. (2008)
*Salvia mirzayanii* Rech. F. & Esfand	↑ 1,8-Cineole, linalyl acetate, α-terpinyl acetate ↓ 5-Neo-cedranol, germacrene D-4-ol, bicyclogermacrene, α-gurjunene	Valifard et al. (2014)
	↑ α-Terpinyl acetate, 1,8-cineole, linalyl acetate	Valifard et al. (2019)
*Salvia officinalis* L.	↑ α-Pinene, camphor, camphene, β-thujone ↓ α-Thujone	Kulak et al. (2020)
	↑ Thujanone, β-pinene, β-myrcene, terpinolene, γ-terpinene ↓ 1,8-Cineole, β-thujone, α-thujone, camphor, β-terpinene, camphene, caryophyllene oxide	Es-sbihi et al. (2021)
	↑ α-Pinene, camphor, camphene ↓ α-Thujone	Kulak et al. (2020)
	↑ α-Thujone, 1,8-cineole, manool ↓ β-Caryophyllene, viridiflorol	Taarit et al. (2009)
*Salvia sclarea* L.	↑ Oxygenated sesquiterpenes, monoterpene hydrocarbons, monoterpene ketones, monoterpene alcohols ↓ Sesquiterpene hydrocarbons, phenols, monoterpene esters, diterpenes, non-terpenes	Taarit et al. (2011)
Phenolic compounds		
*Dracocephalum kotschyi* Boiss.	↑ Total phenolic and flavonoid contents	Vafadar et al. (2020)
*Lavandula angustifolia* Mill.	↓ Total phenolic content	Chrysargyris et al. (2018)
	↑ Total flavonoid content	Szekely-Varga et al. (2020a)
*Lavandula stoechas* L.	↑ Total phenolic content	Valizadeh-Kamran et al. (2019)
	↑ Total flavonoid content	Vojodi Mehrabani et al. (2017)
*Mentha* x *piperita* L.	↑ Total phenolic content	Çoban and Göktürk Baydar (2016)
	↑ Total phenolic content	Khalvandi et al. (2019)
*Mentha pulegium* L.	↑ Total phenolic content	Oueslati et al. (2010)
*Perilla frutescens* L. Britt.	↑ Rosmarinic acid	Rouphael et al. (2019)
*Rosmarinus officinalis* L.	↑ Total phenolic content	El-Esawi et al. (2017)
*Salvia coccínea* Buc’hoz Ex Etl.	↓ Total phenolic content	Grzeszczuk et al. (2018)
*Salvia macrosiphon* Boiss.	↓ Total phenolic content	Valifard et al. (2017)
*Salvia mirzayanii* Rech. F. & Esfand	↑ Total phenolic acid content	Valifard et al. (2014)
*Ocimum basilicum* L.	↓ Rosmarinic acid, chicoric acid, caffeic acid	Bahcesular et al. (2020)
	↑ Phenolic acid content ↓ Anthocyanidins	Bekhradi et al. (2015)
*Ocimum basilicum* L. ‘Sweet Broadleaf’	↑ Quercetin-rutinoside, rosmarinic acid, caffeic acid derivatives, caftaric acid, cinnamyl malic, acid, feruloyl tartaric acid ↓ Chicoric acid, caffeic acid	Scagel et al. (2019)
*Ocimum basilicum* L. ‘Siam Queen’	↑ Caffeic acid derivatives, caftaric acid, cinnamyl malic acid, feruloyl tartaric acid ↓ Chicoric acid	Scagel et al. (2019)
*Origanum majorana* L.	↑ Epigallocatechin, epicatechin, resorcinol, coumarin, quercetin, catechin, quercetin-3-galactoside, apigenin, amentoflavone ↓ Rutin, quercetin	Baâtour et al. (2012)
	↑ Tannin content	Baâtour et al. (2012)
	↑ Gallic acid, caffeic acid, dihydroxyphenolic acid, cinnamic acid, ferulic acid ↓ Rosmarinic acid	Baâtour et al. (2012)
	↑ Total phenolic content	Jelali et al. (2011)
*Origanum vulgare* L.	↑ Gallic acid, caffeic acid, chlorogenic acid, cinnamic acid, quercetin	Azimzadeh et al. (2023)
*Thymus daenensis* Celak	↑ Total phenolic content, gallic acid, rosmarinic acid, chlorogenic acid, rutin, quercetin	Bistgani et al. (2019)
*Thymus vulgaris* L.	↑ Total phenolic content, cinnamic acid, gallic acid, rosmarinic acid, rutin, quercetin	Bistgani et al. (2019)

↑, increase; ↓, decrease.

*Mentha* sp. is another important Lamiaceae genera with a wide range of culinary and aromatic uses, mainly due to their richness in EOs. Aziz et al. (2008) compared the effect of salt stress on the EO chemical composition of pennyroyal (*Mentha pulegium* L.), peppermint (*Mentha piperita*), and apple mint (*Mentha suaveolens* Ehrh.) and observed that the EO yields of the three species decreased compared to the untreated controls. The same occurred in *M. piperita* in another study, where the increase in salinity led to a decrease in EO content (Çoban and Göktürk Baydar, 2016). In hydroponically grown *Mentha spicata* L. plants, the highest amount of NaCl tested by the authors (100 mM) was the only one that improved the EO yield (Chrysargyris et al., 2019b). In hydroponically grown *M. spicata* plants, 150 mM of NaCl did not affect the total EO content, but there was an increase in total monoterpene hydrocarbons and a decrease in total oxygenated monoterpenes (Chrysargyris et al., 2019c).

The use of rosemary (*R. officinalis*) in the Mediterranean region is an attractive solution to avoid desertification and rapid soil erosion due to its great high resistance to environmental conditions (Sarmoum et al., 2019). Bidgoli et al. (2019) found a significant relationship between the percentage of rosemary EO and the increasing salinity stress. More specifically, in another study with *R. officinalis*, salinity altered the major EO compounds, strongly increasing the content of camphor, borneol, linalool, and verbenone, but decreasing β-pinene, α-pinene, and 1,8-cineole (El-Esawi et al., 2017). On the other hand, a 50% decrease in 1,8-cineole content was observed with increasing NaCl concentration (Tounekti et al., 2008). In the case of *Thymus maroccanus* Ball., which behaved as a moderately salt-tolerant species, the EO content did not change with increasing salt concentration (Belaqziz et al., 2009). An important finding was reported by Cordovilla et al. (2014), who showed that plant–plant interactions play a crucial role in stress tolerance. These authors studied the effects of salinity and the interaction between *T. vulgaris* and *L. angustifolia* and found that both plants grown alone were more tolerant to salt stress than when grown together. When exposed to the highest stress intensity (100 mM NaCl), all lavender plants survived, whereas thyme plants died. EO production increased with salinity in *T. vulgaris*, whereas in lavender, it depended on the presence of thyme. Salinity has an obvious effect on *Lavandula stoechas* L. (Vojodi Mehrabani et al., 2017; Valizadeh-Kamran et al., 2019), strongly influencing the EO composition. According to some authors, this could be due to the disruption of cytokine transport from roots to shoots and consequently changes in the ratio of cytokines and abscisic acid (Vojodi Mehrabani et al., 2017). A decrease in EO yield from *M. officinalis* under salt stress was observed although the number of new compounds increased. For example, neryl acetate and geranyl acetate were detected at 100–200 mM of NaCl, suggesting that this species activates the metabolic pathways to produce monoterpenes under salinity (Bonacina et al., 2017).

In addition to EO, salt stress altered the production of PCs by increasing its concentration in the tissues although it limited the biomass production of the plants (Baâtour et al., 2012). The highest levels of PCs were recorded in several Lamiaceae species grown in a medium containing NaCl (Baâtour et al., 2012; Valifard et al., 2014; Vojodi Mehrabani et al., 2017; Valizadeh-Kamran et al., 2019). A recent study investigated the influence of salinity on the production of PCs by two subspecies of *O. vulgare*, namely ssp. *gracile* and *vulgare*. The results indicated an improvement in the contents of quercetin and gallic, caffeic, and chlorogenic acids in ssp. *gracile* and quercetin and caffeic and cinnamic acids in ssp. *vulgare* following salt stress (Azimzadeh et al., 2023). Baâtour et al. (2012) investigated the effect of salinity on phenolics production by *O. majorana* at three developmental stages (early vegetative stage, late or preflowering vegetative stage, and flowering stage). The authors observed that salt stress led to an increase in total and individual phenolic contents (e.g., gallic acid, caffeic acid, and amentoflavone) particularly at the late or preflowering vegetative stage. In the flowering stage, salinity caused a significant stimulation of other phenolic acids (caffeic, syringic, chlorogenic, cinnamic, dihydroxyphenolic, and *trans*-2-hydrocinnamic acids). Furthermore, the degree of salinity stress (moderate or high) significantly influenced the phenolic content of *O. majorana* (Jelali et al., 2011). The results of different studies showed that the positive or negative effects of salinity on basil (*O. basilicum*) depend on the degree of tolerance of the different genotypes. For example, when comparing green Iranian and Genovese basil, salinity stress improved the phenolic acid content only in the former (Bekhradi et al., 2015). In the case of *O. basilicum* ‘Sweet Broadleaf’ and ‘Siam Queen’ cultivars, salt stress increased the leaf content of the two main phenolics (quercetin-rutinoside and rosmarinic acid) only in the ‘Sweet Broadleaf’ cultivar (Scagel et al., 2019). A salt-induced improvement in PC biosynthesis has also been described in *Salvia mirzayanii* (Valifard et al., 2014), *M. piperita* (Çoban and Göktürk Baydar, 2016; Khalvandi et al., 2019), *M. pulegium* (Oueslati et al., 2010), *L. stoechas* (Valizadeh-Kamran et al., 2019), *Dracocephalum kotschyi* Boiss (Vafadar et al., 2020), and *Perilla frutescens* L. Britt (Rouphael et al., 2019). In the case of *L. angustifolia*, flavonoids have been shown to play an important role in salinity tolerance (Szekely-Varga et al., 2020a). On the other hand, in other Lamiaceae species, such as *Salvia coccinea* (Grzeszczuk et al., 2018) and *Salvia macrosiphon* Boiss (Valifard et al., 2017), salinity-exposed plants showed a reduced accumulation of phenolics. Cinnamic acid was the major constituent in *T. daenensis* and *T. vulgaris*, and the concentration of 60 mM NaCl increased its content by more than 30% in both species compared to the control (Bistgani et al., 2019). With regard to rosmarinic acid, the most abundant compound in several Lamiaceae species, the results found in the literature vary, with significant decreases (Baâtour et al., 2012; Bahcesular et al., 2020) or increases (Bistgani et al., 2019; Rouphael et al., 2019; Scagel et al., 2019; Vafadar et al., 2020) in its content observed under salinity stress.

### 4.3 Temperature

Temperature is also one of the most important abiotic factors limiting plant growth and productivity (Israel et al., 2022). Heat and cold stresses affect various physiological, biochemical, and molecular plant characteristics (Mahajan et al., 2020). High temperature can induce leaf senescence and damage to cell membranes, chlorophylls, and proteins (Mansinhos et al., 2022b). Cold stress is also known to be one of the most damaging abiotic stresses, resulting in morphological changes, such as reduced biomass, reduced leaf surface area, leaf curling, and abscission. Biochemically, low temperature stimulates the biosynthesis of ROS, leading to the oxidation of proteins and lipids (Lianopoulou and Bosabalidis, 2014). The transcriptome and metabolism of plants are also significantly affected by cold, probably due to the direct inhibition of metabolic enzymes at low temperatures (Rastogi et al., 2019). Temperature strongly affects the production of secondary metabolites. However, reports on the effect of temperature on the EO content or phenolic profile of aromatic plants remain limited. The studies available in the literature on the effect of temperature stress on the production of EOs and PCs in Lamiaceae species are presented in **Table 3**.

**Table 3:** Alteration in secondary metabolite (essential oils and phenolic compounds) production in Lamiaceae species under temperature stress.

Plant species	Temperature	Reported effect	Reference
Essential oils			
*Lavandula angustifolia* cv. Etherio	29°C	↑ Linalool	Hassiotis et al. (2014)
*Ocimum basilicum* L.	10°C	↑ β-Guaiene, germacrene D, epi-bicyclosesquiphellandrene, terpinolene, α-sesquiphellandrene, γ-cadinene, α-caryophyllene ↓ Geraniol	Senji and Mandoulakani (2018)
*Ocimum tenuiflorum* L.	15°C	↑ Eugenol, methyleugenol, β-caryophyllene	Nguyen et al. (2022)
	4°C	↓ Eugenol, methyleugenol	Rastogi et al. (2019)
*Origanum dictamnus* L.	Winter	↑ ρ-Cymene, borneol, carvacrol ↓ γ-Terpinene, β-caryophyllene, δ-cadinene	Lianopoulou and Bosabalidis (2014)
*Salvia sclarea* L.	2.5°C–3.0°C higher than ambient	↑ Linalyl acetate, spathulenol ↓ Ocimene (Z) beta, linalool, linalyl propionate, geraniol	Kumar et al. (2017)
*Teucrium polium* L.	Winter	↑ Germacrene D, linalool, *trans*-verbenol, α-terpineol, α-ylangene, β-bourbonene, β-caryophyllene, δ-cadinene, spathulenol ↓ β-Pinene	Lianopoulou et al. (2014)
*Thymus transcaucasicus* Ronn.	25°C	↑ α-Terpineol, borneol, limonene, β-myrcene, sabinene ↓ Carvacrol, thymol, linalool, 1,8-cineole	Manukyan (2019)
Phenolic compounds			
*Lavandula viridis* L’Hér	15°C	↑ Rosmarinic acid ↓ Sagerinic acid	Mansinhos et al. (2022b)
	30°C	↑ Rosmarinic acid ↓ Salvianolic acid A, salvianolic acid F, salvianolic acid I	Mansinhos et al. (2022b)
*Mentha spicata* L.	30°C	↓ Rosmarinic acid	Fletcher et al. (2005)
*Ocimum basilicum* L.	55°C	↑ Total phenolic and flavonoid contents	Al-Huqail et al. (2020)
*Rosmarinus officinalis* L.	12°C/6°C	↑ Caffeic acid, carnosic acid, rosmarinic acid ↓ Carnosol	Luis et al. (2007)
*Thymus transcaucasicus* Ronn.	20°C	↑ Chlorogenic acid, apigenin-7-O-glucoside ↓ ρ-Coumaric acid, ferulic acid, rosmarinic acid, quercetin, apigenin, carnosic acid	Manukyan (2019)
*Thymus lotocephalus* López and Morales	15°C	↑ Rosmarinic acid, methyl 6-O-galloyl-β-D-glucopyranoside, caffeic acid ↓ Sagerinic acid, methylrosmarinic acid isomer II, salvianolic acid B	Mansinhos et al. (2022b)
	30°C	↑ Rosmarinic acid, salviaflaside, methyl 6-O-galloyl-β-D-glucopyranoside, salvianolic acid I, salvianolic acid C, salvianolic acid A ↓ Sagerinic acid, salvianolic acid B	Mansinhos et al. (2022b)

↑, increase; ↓, decrease.

Some studies have shown that EO composition is affected by low temperatures. For example, *O. tenuiflorum*, an *Ocimum* species that grows well in tropical, subtropical, and warm temperate regions, was exposed to cold stress (4°C) for 5 days. After this period, the eugenol content increased from 17.7 to 45.2 mg/g_FW_ at the juvenile stage (30 days old). A significant improvement in methyleugenol and β-caryophyllene content was also observed at the juvenile and preflowering stages (Nguyen et al., 2022). On the other hand, other authors reported that eugenol and methyl eugenol concentrations in *O. tenuiflorum* EO decreased under cold conditions (Rastogi et al., 2019). *O. basilicum* plants were exposed to different temperatures of 4°C, 10°C, and 22°C (control) for 12 h, 24 h, and 48 h, and significant differences between treatments were found for almost all EO compounds. Linalool was the main compound, but there were no differences between the cold treatments and the control. On the contrary, the highest amount of the sesquiterpene germacrene D (the second most abundant compound) was found at 10°C for 12 h. In the case of geraniol and γ-cadinene, their maximum levels were observed at 4°C for 24 h (Senji and Mandoulakani, 2018). Lianopoulou and Bosabalidis (2014) compared the EO composition of *Origanum dictamnus L.* in winter and summer and observed that in summer, these plants produced more than twice the EO yield at higher temperatures than at lower temperatures. According to the authors, this higher EO yield in summer is correlated with the greater number of peltate glandular hairs found on summer leaves. This means that peltate hairs (and especially their head cells) are the only cells of all leaf tissues that possess the required enzymatic apparatus required for EO biosynthesis. Regarding the EO profile, there was also a variation in the main compounds, with the most abundant compound being ρ-cymene (59.2%) in winter and carvacrol (42.4%) in summer. Interestingly, the same authors repeated the same study on another Lamiaceae species, *Teucrium polium* L., and observed the opposite results, a significantly higher EO yield of winter leaves compared to summer leaves. Nevertheless, the authors again correlated the higher EO yield with the higher number of peltate glandular hairs in winter leaves (Lianopoulou et al., 2014). The maximum content and yield of *Thymus transcaucasicus* Ronn. EO were observed at 25°C compared to 15°C and 20°C (Manukyan, 2019).

There are some studies in the literature related to heat stress. Plant extracts of *R. officinalis* are routinely used for food preservation and cooking, cosmetics, or in herbal medicine for anti-inflammatory and antimicrobial purposes, as well as for the treatment of diabetes and cardiovascular disorders (Degner et al., 2009). Two accessions of this species were exposed to 12°C/6°C day/night, and both accessions showed an increase in carnosic and caffeic acid levels, while rosmarinic acid, carnosol, naringin, hispidulin, and cirsimaritin showed different responses (Luis et al., 2007). Elevated temperature (2.5°C–3.0°C above ambient) had a significant effect on the EO composition of *S. sclarea*, especially for the abundant linalyl acetate, almost doubling its concentration (Kumar et al., 2020). Similarly, the pattern of the monoterpene linalool, the most abundant component of the EO of *L. angustifolia*, was affected by temperature (Hassiotis et al., 2014). Linalool is widely used as an additive in 60%–80% of perfumed hygiene and cleaning products such as detergents, lotions, soaps, and shampoos and as an insecticide against fruit flies, cockroaches, and fleas (Senji and Mandoulakani, 2018). Some reports were found in the literature regarding the influence of temperature on PC biosynthesis. Recently, the effect of different temperature treatments (15°C, 20°C, 25°C, and 30°C) on the phenolic profile of *in vitro* cultures and micropropagated plants of two Mediterranean aromatic plants, *T. lotocephalus* and *Lavandula viridis* L’Hér, was evaluated. For both species, the production of phenolic acids (especially rosmarinic acid) increased with increasing temperature in micropropagated plants, whereas the opposite was observed in *in vitro* cultures (Mansinhos et al., 2022b). Another study also investigated the effect of constant air temperature (15°C, 20°C, and 25°C) on phenolic production of *T. transcaucasicus* plants grown in pots, and the temperature of 25°C was the most favorable for the best recovery of rosmarinic acid (Manukyan, 2019). Temperature stress led to a significant increase in total phenolics and flavonoids in basil (*O. basilicum*) leaves subjected to the highest temperature treatment (55°C) (Al-Huqail et al., 2020). In spearmint (*M. spicata*), heat significantly reduced the levels of total phenolic acids and especially rosmarinic acid (Fletcher et al., 2005). A high accumulation of rosmarinic acid was also obtained in *M. officinalis* under heat stress (38°C for 5 h) (Pistelli et al., 2019).

### 4.4 Light

The three main factors of light that strongly affect plant growth and other physiologic, biochemical, and metabolic processes are quality, quantity, and duration (Israel et al., 2022; Gonçalves et al., 2023). Despite the importance of light in several plant processes, it can also act as a stress factor. Secondary metabolites play an essential role in coping with light stress. The impact of light stress on the biosynthesis of EO and PC in Lamiaceae species is presented in **Table 4**. The spectral quality of light is a common parameter that is typically accessed through the use of light-emitting diodes (LEDs). For example, *O. basilicum*, one of the most extensively studied species within the Lamiaceae family, was cultivated under four distinct monochromatic light sources, each of which was irradiated with a specific wavelength: blue (470 nm), blue-green (500 nm), green (525 nm), and red (660 nm). After 70 days, the authors observed that the greatest concentration of EO was observed in the leaves grown under blue light and that 1,8-cineole was the most abundant compound, irrespective of light quality (Amaki et al., 2011). Similarly, *P. abrotanoides* and *P. atriplicifolia* were subjected to four distinct artificial light treatments (red, blue, red-blue, and white) generated by LEDs. The results indicated that the highest wavelengths caused a reduction and an increase in monoterpene and sesquiterpene contents, respectively (Ghaffari et al., 2019). The production of EOs from other Lamiaceae species, including *M. piperita*, *M. spicata*, and *M. longifolia*, was enhanced by the application of LED treatments (Sabzalian et al., 2014).

**Table 4:** Alteration in secondary metabolite (essential oils and phenolic compounds) production in Lamiaceae species under light stress.

Plant species	Light	Reported effect	Reference
Essential oils			
*Aeollanthus suaveolens* Mart. ex Spreng	Solar irradiance (50%)	↑ (E)-β-Farnesene, β-elemene, *cis*-α-bergamotene, α-santalene ↓ Camphene, β pinene, myrcene, linalool, geranyl formate	Barbosa et al. (2021)
*Melissa officinalis* L.	PAR and UV-B radiation	↑ Geranial, neral, 6-methyl-5-hepten-2-on ↓ Citronellol, geraniol, citronellal	Manukyan (2013)
	Solar irradiance	↑ Neral, geranial, methyl geranate, E-caryophyllene	Hosseinabadi et al. (2024)
*Mentha longifolia* L.	LED (red-blue)	↑ EO yield	Sabzalian et al. (2014)
*Mentha piperita* L.	LED (red)	↑ EO yield	Sabzalian et al. (2014)
*Mentha spicata* L.	LED (blue)	↑ EO yield	Sabzalian et al. (2014)
*Nepeta cataria* L.	PAR and UV-B radiation	↑ Citronellol, β-caryophyllene ↓ *trans*-3-Hexen-1-ol, linalool, neral, geranial	Manukyan (2013)
*Ocimum basilicum* L.	Solar irradiance	↑ Eugenol, linalool, methyl eugenol	Chang et al. (2008)
	UV-B radiation	↑ 1,8-Cineole, linalool, α-pinene, β-pinene, β-ocimene ↓ α-Terpinene, eugenol	Chang et al. (2009)
	LEDs (blue)	↑ 1,8-Cineole, α-pinene, β-pinene, myrcene, limonene, γ-terpinene, linalool	Amaki et al. (2011)
*Ocimum sanctum* L.	UV-B radiation	↑ β-Elemene, germacrene D, β-caryophyllene ↓ β-Selinene	Kumari and Agrawal (2011)
*Perovskia abrotanoides* Kar.	LEDs (blue, red-blue, red)	↑ δ-3-Carene, α-humulene, *trans*-caryophyllene ↓ α-Pinene, camphene, 1,8-cineole, camphor, borneol, bornyl acetate	Ghaffari et al. (2019)
*Perovskia atriplicifolia* Benth.	LEDs (blue, red-blue, red)	↑ Camphene, δ-3-carene, 1,8-cineole, *trans*-caryophyllene, α-humulene ↓ α-Pinene, bornyl acetate	Ghaffari et al. (2019)
*Rosmarinus officinalis* L.	Darkness	↑ Caryophyllene oxide ↓ 1,8-Cineole, camphene, α-pinene, β-terpinene, camphor	Böszörményi et al. (2020)
	Darkness (50%)	↑ *α*-Pinene, camphene, *β*-caryophyllene, *β*-pinene, myrcene ↓ 3-Carene, ρ-cymene, camphor, borneol, verbenone	Raffo et al. (2020)
*Salvia officinalis* L.	PAR and UV-B radiation	↑ Camphene, *α*-terpinene, camphor ↓ Manool, viridiflorol, *α-*humalene*, β*-pinene	Manukyan (2013)
Phenolic compounds			
*Agastache rugosa* (Fischer & C.A. Meyer) O. Kuntze	LEDs (white/PAR)	↑ Rosmarinic acid	Zielińska et al. (2019)
*Melissa officinalis* L.	LEDs (red:blue:green:far-red, 40%:35%:15%:10%)	↑ Rosmarinic acid	Chai et al. (2023)
	PAR and UV-B radiation	↑ Rosmarinic acid, caffeic acid, melitic acid	Manukyan (2013)
*Moluccella laevis* L.	PAR	↑ Ferulic acid, rosmarinic acid, caffeic acid	Zielińska et al. (2020)
*Nepeta cataria* L.	PAR and UV-B radiation	↑ Rosmarinic acid, caffeic acid ↓ ρ-Coumaric acid	Manukyan (2013)
*Nepeta nuda* L.	LEDs (blue:red:far-red, 15%:75%:10%)	↑ Rosmarinic acid, chlorogenic acid, cirsimaritin, rutin, isoquercetin ↓ Ferulic acid	Petrova et al. (2022)
*Ocimum basilicum* L.	UV-B radiation	↑ Total phenolic and anthocyanin contents	Sakalauskaitė et al. (2012)
*Plectranthus scutellarioides* L.	Ceramic metal halide lamp (CDM)	↑ Total phenolic, anthocyanin and flavonoid contents, rosmarinic acid, luteolin hexoside, luteolin glucuronide, apigenin-X	Dörr et al. (2019)
*Perovskia abrotanoides* Kar.	LEDs (blue)	↑ Total phenolic and flavonoid contents	Ghaffari et al. (2019)
	LEDs (white, red, red-blue)	↓ Total phenolic and flavonoid contents	Ghaffari et al. (2019)
*Perovskia atriplicifolia* Benth.	LEDs (blue, red, white)	↑ Total phenolic and flavonoid contents	Ghaffari et al. (2019)
*Rosmarinus officinalis* L.	UV-B radiation	↑ Rosmarinic acid, carnosic acid, carnosol, naringin, caffeic acid, cirsimaritin, hispidulin	Luis et al. (2007)
*Salvia officinalis* L.	PAR and UV-B radiation	↑ Rosmarinic acid, caffeic acid, melitic acid	Manukyan (2013)
*Satureja hortensis* L.	LEDs (blue, red, green)	↓ Total flavonoid content	Khlebnikova et al. (2022)
*Scutellaria baicalensis* Georgi	UV-B radiation	↑ Chrysin ↓ Scutellarin	Tang et al. (2014)
*Scutellaria lateriflora* L.	Darkness	↑ Verbascoside, scutellarin ↓ Scutellarein, baicalein	Marsh et al. (2014)

↑, increase; ↓, decrease.

The impact of UV radiation on plants is a topic that has been extensively researched. Although the Earth’s surface only receives 20% of UV-B radiation, this energy provides sufficient energy to cause several biological injuries to plants, including damage to DNA. For this reason, UV-B is the most extensively studied type of UV radiation (Mansinhos et al., 2024). The plant photoreceptors of plants respond to UV-B radiation, regulating plant growth and metabolism. This increases the plant’s capability to protect itself from the harmful consequences of UV radiation (Hosseinabadi et al., 2024). Chang et al. (2008) investigated the impact of solar irradiance and UV-B radiation (Chang et al., 2009) on *O. basilicum*, demonstrating a positive effect of both treatments on linalool, a key taste compound in basil. Similarly, *O. sanctum* was subjected to UV-B radiation. While the content of the EO major compound (eugenol) was not affected by the treatment, the EO yield increased by 42% (Kumari and Agrawal, 2011). The analysis of volatile oils in fresh leaf samples of basil demonstrated that UV-B irradiation also stimulated the synthesis of 1,8-cineole and linalool. In contrast, no effect was observed on the volatile composition of the EO. The (partial) absence of light can also serve as a stimulus for the production of bioactive compounds. *Aeollanthus suaveolens*, popularly known as Catinga-de-mulata, is employed by the Amazonian population for the treatment of pain. The chemical profile of this medicinal plant was studied in response to two light intensities, namely, 50% and 100% of sunlight. It was observed that the concentration of the most abundant compound (E)-β-farnesene was increased by half shade treatment (Barbosa et al., 2021). Additionally, intermediate shading (50% of sunlight) demonstrated a beneficial impact on the yield of *R. officinalis* EO, resulting in an increase of 29% compared to full solar irradiance (Raffo et al., 2020). Conversely, the impact of prolonged darkness on the composition of EO from this species was examined, and no discernible differences in monoterpenes and sesquiterpenes were observed between the light- and dark-grown leaves (Böszörményi et al., 2020). In a recent study, Hosseinabadi et al. (2024) examined the impact of varying harvesting times on the yield of EO from *M. officinalis.* Their findings indicated a positive correlation between the increase in UV-B levels during the summer months and the EO yield. The influence of photosynthetically active radiation (PAR) and UV-B radiation on the EO of sage (*S. officinalis*), lemon balm (*M. officinalis*), and lemon catmint (*Nepeta cataria* L.) was investigated by Manukyan (2013). The highest yield and content of EO were observed in *N. cataria*, with both parameters significantly enhanced by both radiations. In the case of *S. officinalis*, no significant differences were observed. In contrast, the radiation displayed a negative effect on the EO content of *M. officinalis*.

The utilization of spectrally tunable LEDs for elicitation has also demonstrated promising potential for augmenting PC production in several species, including *M. officinalis* (Chai et al., 2023), *Plectranthus scutellarioides* L (Dörr et al., 2019), *Agastache rugosa* (Fischer & C.A. Meyer) O. Kuntze (Zielińska et al., 2019), and *Nepeta nuda* L. (Petrova et al., 2022). In a recent study, Chai et al. (2023) evaluated the impact of three distinct light treatments on rosmarinic acid production. The treatments included a) three different spectrally tuned light treatments, b) white LED, and c) sunlight. The optimal result was achieved with the blue-enriched light treatment (red:blue:green:far-red, 40%:35%:15%:10%). Additionally, the leaves of *P. scutellarioides* grown with a ceramic metal halide lamp (CDM) and LED light exhibited the highest concentration of rosmarinic acid (Dörr et al., 2019).

In addition to the type of light, the intensity of light also influences the production of PC. In the case of *N. nuda*, the high light intensity of LEDs (blue:red:far-red, 15%:75%:10%) stimulated the production of PC-like compounds, including rosmarinic acid, isoquercetin, rutin, cirsimaritin, and naringenin. Conversely, a lower intensity significantly upregulated the production of chlorogenic acid (Petrova et al., 2022). With regard to ultraviolet radiation, there is substantial evidence indicating the considerable impact of UV-B radiation on the production of PC (Luis et al., 2007; Sakalauskaitė et al., 2012; Tang et al., 2014). *O. basilicum* was subjected to two different intensities of UV-B radiation (2 kJ m^−2^ day^-1^ and 4 kJ m^−2^ day^-1^) for 7 days. A comparison with the control revealed that the total PC levels were significantly higher in both UV treatments (Sakalauskaitė et al., 2012). The application of UV-B radiation resulted in an increase in the concentrations of rosmarinic and carnosic acids in rosemary plants, as well as naringin and carnosol (Luis et al., 2007). Similarly, the use of different UV-B light intensities was found to activate chrysin synthesis in *S. baicalensis* L., which was not detected in the control plants (Tang et al., 2014). The impact of UV-B radiation and PAR on PC production was also investigated in *S. officinalis*, *M. officinalis*, and *N. cataria*. The application of PAR treatment was found to be conducive to PC accumulation in *M. officinalis*, while intensive UV-B radiation was observed to stimulate the highest PC production in *S. officinalis* (Manukyan, 2013). Furthermore, PAR enhanced the biosynthesis of the phenolic acids rosmarinic, caffeic, and ferulic in *Moluccella laevis* L (Zielińska et al., 2020).

### 4.5 Heavy metals

The accumulation of heavy metals (HMs) in the soil, air, and water is influenced by a number of factors, including metal industries, mining, and intensive agriculture. These activities can lead to the accumulation of essential HMs, such as copper (Cu), iron (Fe), zinc (Zn), and magnesium (Mg), which are required for plant growth and development. However, they can also result in the accumulation of non-essential HMs, such as lead (Pb), nickel (Ni), arsenic (As), mercury (Hg), chromium (Cr), and cadmium (Cd), in excess of acceptable limits (Mishra and Chandra, 2022; Gonçalves et al., 2023; Das et al., 2024). Soil contamination with HMs represents a significant environmental concern, with significant implications for human health. HMs can disrupt the normal functioning of the organs, leading to a range of illnesses, including abdominal pain, skin eruptions, ulcers, and cancer (Jezler et al., 2015). Furthermore, HMs are a significant threat to plant health, impeding photosynthesis, respiration, and nutrient uptake. The literature indicates that essential HMs are harmful to plants when present in high amounts, whereas non-essential metals are particularly injurious even when present in small concentrations (Mishra and Chandra, 2022; Das et al., 2024). In order to mitigate the effects of free radicals associated with HM stress, plants typically accumulate elevated levels of secondary metabolites that function as antioxidant agents (Gonçalves et al., 2023). **Table 5** presents a summary of the effects of HM stress on the production of EOs and PCs of MAPs from the Lamiaceae family. A number of studies have been conducted in the literature which report the impact of HMs on the EOs of *Ocimum* spp. (Rai et al., 2004; Zheljazkov et al., 2006; Prasad et al., 2011; Siddiqui et al., 2013; Fattahi et al., 2019). The composition of the EOs of *O. basilicum* was found to be altered by the presence of Ni, Cr, Cd, and Pb, with the type and concentration of the HMs influencing the extent of the changes (Prasad et al., 2011). Fattahi et al. (2019) reported that Cd and Pb stimulated the EO yield and induced a positive influence on EO composition, with a notable impact on the production of its most abundant compound estragole. Similarly, Cd and Pb (individually and in combination) enhanced the limonene and 1,8-cineole contents in *O. basilicum* EOs, yet had no impact on the content of their major compounds menthol and menthone (Zheljazkov et al., 2006). *O. tenuiflorum* L. was subjected to a range of Cr concentrations (10, 20, 50, and 100 mM), which resulted in the production of eugenol, its major compound, particularly at 20 mM (Rai et al., 2004). Lower doses of As induced the accumulation of the major compounds (eugenol, methyl chavicol, and linalool) of *O. tenuiflorum*, *O. basilicum*, and *O. gratissimum* L. EOs. It is encouraging to note that no evidence of As was found in the EOs of any species, which makes these EOs suitable for industrial applications (Siddiqui et al., 2013).

**Table 5:** Alteration in secondary metabolite (essential oils and phenolic compounds) production in Lamiaceae species under heavy metal stress.

Plant species	Heavy metals	Reported effect	Reference
Essential oils			
*Mentha arvensis* L.	Lead	↑ Limonene, isomenthone	Jezler et al. (2015)
	Cadmium	↑ Limonene, isomenthone	Jezler et al. (2015)
	Chromium	↑ Limonene, menthone, menthyl acetate, menthol ↓ α-Pinene, β-pinene, β-myrcene	Prasad et al. (2010)
	Lead	↑ Limonene, menthone, menthol	Prasad et al. (2010)
*Mentha citrata* Ehrh.	Chromium	↓ (Z)-β-Ocimene	Prasad et al. (2010)
	Lead	↑ Linalool, linalyl acetate ↓ β-Myrcene, β-pinene, sabinene, limonene, (Z)-β-ocimene	Prasad et al. (2010)
*Mentha crispa* L.	Lead	↑ Carvone, limonene ↓ Linalool, (+)-*trans*-carveol, bornyl acetate, β-cariophyllene, *trans*-β-farnesene, germacrene D	Sá et al. (2015)
*Melissa officinalis* L.	Cadmium	↓ EO content	Kilic and Kilic (2017)
*Mentha* x *piperita* L.	Cadmium	↑ Menthofuran, pulegone ↓ Menthol	Azimychetabi et al. (2021)
		↑ 1,8-Cineole, β-caryophyllene, α-pinene, limonene ↓ Menthofuran, menthyl acetate	Zheljazkov et al. (2006)
	Lead	↑ 1,8-Cineole, limonene, α-pinene, menthyl acetate, β-caryophyllene, pulegone ↓ Menthone, menthol, neomenthol	Zheljazkov et al. (2006)
		↑ Menthol ↓ Menthofuran, menthonel, pulegone	Safarian et al. (2023)
		↑ Methyl acetate, menthol menthoman ↓ α-Pinene, β-pinene, sabinene, limonene, menthone	Prasad et al. (2010)
	Copper	↑ Limonene ↓ Menthol, menthone, α-pinene, neomenthol, menthofuran	Zheljazkov et al. (2006)
	Chromium	↑ Menthone ↓ α-Pinene, β-pinene, sabinene, limonene, 1,8-cineole	Prasad et al. (2010)
*Mentha pulegium* L.	Copper	↑ Thymol, 1,8-cineole, α-pinene, pulegone, *cis*-isopulegone, sabinene	Lajayer et al. (2017)
*Ocimum basilicum* L.	Arsenite	↑ Linalool, eugenol, methyl eugenol, methyl chavicol, β-caryophyllene, β-ocimene	Siddiqui et al. (2013)
	Cadmium	↑ Estragole, octanol, linalool, oxide γ-cadinene, nerol, neryl acetate, caryophyllene ↓ 1,8-Cineole, β-selinene, α-pinene, geranial, *cis*-α-bisabolene, α-thujene methyl eugenol	Fattahi et al. (2019)
	Lead, chromium, cadmium	↑ Methyl chavicol, methyl eugenol ↓ Linalool	Prasad et al. (2011)
	Nickel	↑ Methyl eugenol ↓ Methyl chavicol, linalool	Prasad et al. (2011)
	Lead	↑ Linalool, estragole, octanol, nerol, neryl acetate, neophytadiene, caryophyllene oxide, oxabicyclododeca ↓ 1,8-Cineole, β-pinene, methyl eugenol, octanal, α-pinene, caryophyllene, geranial, β-bisabolene, geranyl acetate, β-selinene	Fattahi et al. (2019)
*Ocimum gratissimum* L.	Arsenite	↑ Eugenol, 1,8-cineole, germacrene-D, β-ocimene	Siddiqui et al. (2013)
*Ocimum tenuiflorum* L.	Arsenite	↑ Eugenol, carvacrol, β-caryophyllene, methyl eugenol, methyl chavicol	Siddiqui et al. (2013)
	Chromium	↑ Eugenol	Rai et al. (2004)
*Rosmarinus officinalis* L.	Lead, arsenic, zinc, and copper	↑ Sesquiterpenes	Affholder et al. (2013)
*Salvia officinalis* L.	Lead, cadmium, zinc, and copper	↑ Camphor, borneol, 1,8-cineole, bornylacetate ↓ α-Thujon, β-thujon, β-cariophyllene, viridoflorol	Stancheva et al. (2009)
Phenolic compounds			
*Basilicum polystachyon* (L.) Moench	Mercury	↑ Vanillic acid, ellagic acid, rosmarinic acid, caffeic acid ↓ *trans*-Cinnamic acid	Das et al. (2024)
	Lead	↑ Caffeic acid, ρ-coumaric acid, ellagic acid, rosmarinic acid ↓ *trans*-Cinnamic acid	Das et al. (2024)
*Ocimum basilicum* L.	Cadmium	↑ Total phenolic and flavonoid contents, rosmarinic acid, chicoric acid, rutin, quercetin	Açıkgöz (2020)
*Origanum vulgare* L.	Zinc	↓ Total phenolic content	Kulbat-Warycha et al. (2020)
	Copper and nickel	↑ Total phenolic content	Kulbat-Warycha et al. (2020)

↑, increase; ↓, decrease.

*Mentha* is another Lamiaceae genus extensively studied in terms of EO production in response to HM stress (Zheljazkov et al., 2006; Prasad et al., 2010; Affholder et al., 2013; Jezler et al., 2015; Sá et al., 2015; Kilic and Kilic, 2017; Lajayer et al., 2017; Azimychetabi et al., 2021). The application of Cu (5 mg kg^−1^) and Zn (10 mg kg^−1^) (Lajayer et al., 2017) resulted in an increase in the yield and content of EO in *M. pulegium*. The presence of Cd (10–40 ppm) was found to decrease the major compound, menthol, while simultaneously increasing the menthofuran and pulegone contents in *M. piperita* EO (Azimychetabi et al., 2021). In a recent study, Safarian et al. (2023) demonstrated that the relative abundance of the enzymes menthol reductase and pulegone reductase were upregulated in *M. piperita*, in contrast to menthofuran synthase, which was downregulated during Pb stress. Furthermore, the authors demonstrated the crucial role of menthol in mitigating the effects of this environmental stress. In *Mentha arvensis* L., the cellular alterations induced by HM application (Pb and Cd, 8–128 mg kg^−1^) were insufficient to produce phytotoxicity in the plant or modify the EO yield (Jezler et al., 2015). A pot culture experiment was conducted to investigate the impact of two concentrations (30 and 60 mg kg^−1^) of Cr and Pb on the yield and chemical composition of EOs from three plant species: marjoram (*M. arvensis*), bergamot mint (*M. citrata* Ehrh.), and peppermint (*M. piperita*). The EO content of the three species decreased with both concentrations of both metals but increased EO yield in *M. piperita*. The effect of increasing concentrations of Pb (900, 1,800, 3,600, 7,200, and 9,000 mg kg^−1^) on the yield and chemical composition of EO of garden mint (*Mentha crispa* L.) was investigated. The EO yield was approximately 10-fold higher under extreme contamination conditions compared to that produced by the control. Moreover, although the majority of compounds were not detected under the Pb context, the content of its major component (carvone) increased from 39.3% (control) to 85.32%–95.04% after growth in Pb-contaminated soils (Sá et al., 2015). The presence of Cd in the soil negatively influenced the EO content in *M. officinalis* L. subsp. *officinalis*. The highest Cd concentration (30 mg kg^−1^) resulted in a 97% decrease in EO content (Kilic and Kilic, 2017). Conversely, an increased biosynthesis of sesquiterpenes in EO was observed in rosemary plants growing in contaminated regions with Pb, As, Zn, and Cu (Affholder et al., 2013). The presence of heavy metals, including Cd, Zn, Cu, and Pb, in the environment resulted in a reduction in the concentrations of α-thujon, β-thujon, viridoflorol, and β-cariophyllene in the EOs of *S. officinalis*. Conversely, the levels of 1,8-cineole, camphor, bornylacetate, and borneol were significantly enhanced (Stancheva et al., 2009).

A limited number of studies were identified in the literature that examined the influence of HMs on the biosynthesis of PCs in Lamiaceae species. *Basilicum polystachyon* (L.) Moench, a MAP found in Asia, Africa, and India, was cultivated *in vitro* with mercury (II) chloride and lead (II) nitrate. In cultures exposed to Hg and Pb, the esterified forms of vanillic acid and caffeic acid were respectively the major PCs, which were not detected in the control (Das et al., 2024). In a further *in vitro* study, using cell suspension cultures, *O. basilicum* was exposed to different concentrations (5–200 µM) of cadmium chloride (CdCl_2_) (Açıkgöz, 2020). The highest contents of rosmarinic and chicoric acids were achieved at 5 µM, while the highest accumulation of the flavonoids rutin and quercetin was observed at 10 µM. Finally, the effect of three HMs (Ni, Cu, Zn), at distinct concentrations, was studied in *O. vulgare.* It was observed that increasing levels of Zn and Cu caused a decrease in the PC concentration (Kulbat-Warycha et al., 2020).

## 5 Effect of environmental stress factors on biological properties and applications

Plants from the Lamiaceae family are known for their strong antioxidant activity, mainly due to their high PC content, which is widely used in health supplements and food preservation (Baâtour et al., 2012). In addition to antioxidant activity, plants belonging to this family have other important biological properties, such as anti-inflammatory, antitumoral, antibacterial, and antiviral, among others, which made them very interesting for researchers, food producers, and consumers (Grzeszczuk et al., 2018). Nevertheless, as concluded from the above data, the production of EOs and PCs from many Lamiaceae species is strongly influenced by environmental conditions, and the accelerating context of climate change may affect the future production and use of these species in various industries. However, most studies only focus on the effect on the percentage/amount of constituents, and concrete details on the effect on the biological properties are lacking. For example, there are few studies in the literature on the effects of drought, salinity, temperature, light, and heavy metals on the biological properties (mostly antioxidant activity) of Lamiaceae species.

The overproduction of ROS leading to oxidative stress is known to be induced by abiotic stresses. In this context, plants are provided with an antioxidant defense system, including enzymatic and non-enzymatic ones, to counteract oxidative stress. According to the literature, the radical scavenging properties of the plant extracts have been shown to be involved in abiotic stress tolerance, and most of the studies are related to osmotic stress, namely, drought and salinity. *Salvia* is the largest genus in the Lamiaceae family with over 900 species. Many have been used in traditional medicine for centuries as antiseptics, antispasmodics, antirheumatics, astringents, antihydrotics, tonics, antimicrobials, carminatives, digestives, anti-inflammatories, and chronic painkillers. Many reports have identified *Salvia* as one of the most important sources of natural antioxidants that help to maintain health and protect against various diseases, such as diabetes, stroke, cancer, atherosclerosis, and neurodegenerative diseases (Grzeszczuk et al., 2018). In response to different abiotic stresses, namely, salinity in the case of *S. mirzayanii* (Valifard et al., 2014) and *S. macrosiphon* (Valifard et al., 2017) and drought in *S. fruticosa* (Chrysargyris et al., 2016), the extracts of these plants exhibited increased antioxidant activity. Similarly, Chrysargyris et al. (2019a) studied the effects of drought in another Lamiaceae plant (*S. perfoliata*) and observed that deficit irrigation enhanced the antioxidant properties of plant extracts. Conversely, water scarcity was found to significantly reduce the antioxidant activity of *S. dolomitica* (Caser et al., 2019) and *S. sinaloensis* (Caser et al., 2018) extracts accessed by the ferric reducing antioxidant power (FRAP) method. *O. basilicum* is a highly valuable annual herb that is commercially cultivated in numerous countries worldwide. It has been reported that this species has several biological properties, including antimicrobial, antioxidant, anti-inflammatory, analgesic, anaesthetic, anti-ulcerogenic, anti-tuberculosis, and cardiac and immune-stimulant effects (Tamokou et al., 2017; Açıkgöz, 2021). *O. basilicum* plants were subjected to reduced irrigation, and a positive effect on the antioxidant capacity of their extract was observed (Pirbalouti et al., 2017). In contrast, salt stress was found to reduce the antioxidant activity of *O. basilicum* extracts (Bahcesular et al., 2020). In *Mentha* species, the impact of osmotic stress is also variable. The DPPH˙ scavenging potential of *M. pulegium* was found to be significantly elevated in the leaves under salinity conditions, whereas the scavenging potential of the root extracts remained unaltered (Oueslati et al., 2010). Chrysargyris et al. (2019c) observed that exposure of *M. spicata* plants to 150 mM of NaCl did not affect the antioxidant capacity, as assessed by the FRAP, DPPH˙, and ABTS˙ methods. Conversely, in *M. piperita* (Khalvandi et al., 2019), *R. officinalis* (El-Esawi et al., 2017; Mohammadzadeh and Pirzad, 2021), *T. daenensis* (Bistgani et al., 2019), *T. vulgaris* (Khalil et al., 2018; Bistgani et al., 2019; Razavizadeh et al., 2019; Mohammadzadeh and Pirzad, 2021), and *D. kotschyi* (Vafadar et al., 2020), the capacity of plant extracts to scavenge free radicals has been demonstrated to improve with increasing osmotic stress induced by salinity or drought. In several cases and as demonstrated above, osmotic stress induced by climate change factors stimulates the antioxidant activity of plant extracts, but sometimes the opposite effect is observed. Lavender is an example of a cultivated plant, either as an ornamental or for EO production and pharmaceutical purposes, and is widely used in medicines, perfumes, balms, lotions, and other cosmetics. *L. angustifolia* was exposed to 25, 50, and 100 mM of NaCl, and the antioxidant capacity (FRAP, DPPH˙, and ABTS˙) of its extracts significantly decreased with the increasing stress intensity (Chrysargyris et al., 2018). The results from other Lamiaceae spp. such as *O. majorana* (Baâtour et al., 2012), *S. coccinea* (Grzeszczuk et al., 2018), *P. atriplicifolia*, and *P. abrotanoides* (Afshari et al., 2022) demonstrated a negative effect on the antioxidant activity of plant extracts when exposed to salt stress or drought conditions in *T. lotocephalus* (Mansinhos et al., 2022a). Temperature also has a major effect on the antioxidant activity of the plant extracts. *T. transcaucasicus* plants were exposed to 15°C, 20°C, and 25°C, and the strongest antioxidant activity was observed at 20°C (Manukyan, 2019). Similarly, *T. lotocephalus* and *L. viridis* were grown at 15°C, 20°C, 25°C, and 30°C for 2 weeks, but the highest antioxidant activities were achieved at the extreme temperatures (15°C and 30°C) (Mansinhos et al., 2022b). On the other hand, after exposure of *M. spicata* to 30°C, the plant lost 21%–60% of its total antioxidant capacity after week 1 and up to 95% by week 4 (Pistelli et al., 2019).

A study by Sakalauskaitė et al. (2012) found that plant extracts from basil plants exposed to UV-B radiation exhibited a significantly higher activity in quenching DPPH radicals in comparison to non-treated plants. Furthermore, in a more recent study, the authors evaluated the impact of different spectrally tuned light treatments on the antioxidant activity of *M. officinalis* leaf extracts. The leaves harvested from the 40% red, 35% blue, 15% green, and 10% far-red treatments evidenced the strongest activity (Chai et al., 2023). In the context of HM pollution, increasing concentrations of Cu and Zn were found to result in a reduction in the antioxidant capacity of *O. vulgare* extracts, as assessed by the FRAP assay (Kulbat-Warycha et al., 2020). Conversely, the greatest enhancements in the radical scavenging capacity of *O. basilicum* were observed in the presence of 5 μM of CdCl_2_.

Although less studied, the antioxidant capacity of EO is also affected by climate change conditions. This is potentially important given the advantages of EOs over synthetic antioxidants, which often have negative health effects, to be used as alternative additives for the food industry and medicine (Tit and Bungau, 2023). Pirbalouti et al. (2014) demonstrated that the antioxidant activity of the EOs from *T. daenensis* plants cultivated under normal irrigation conditions was significantly higher than that observed under limited irrigation. Although Cd (10–40 ppm) significantly decreased the major compound of *M. officinalis* EO (menthol), it did not compromise its antioxidant properties, which increased with the increasing concentration of Cd stress (Azimychetabi et al., 2021). Similarly, there was a dose-dependent increase in the antioxidant capacity of peppermint, with the highest value obtained at 600 ppm of Pb (Safarian et al., 2023).

Few studies were found in the literature on the effect of abiotic stress on other biological properties of Lamiaceae species (beyond antioxidant activity). The antibacterial activity of *R. officinalis* EO against *Listeria monocytogenes*, *Bacillus cereus*, *Staphylococcus aureus*, *Micrococcus flavus*, *Escherichia coli*, and *Pseudomonas aeruginosa* was improved with increasing salt concentration. According to the authors, these activities were much higher than the antibiotics tested (El-Esawi et al., 2017). Similarly, the EO of *O. majorana* exposed to saline conditions (75 mM of NaCl) showed higher antibacterial activity (especially against Gram-negative bacteria) compared to plants grown under normal conditions (Olfa et al., 2016). The extracts from *M. officinalis* plants subjected to LEDs (red:blue:green:far-red, 40%:35%:15%:10%) were assessed against three Gram-positive bacteria (*S. aureus*, methicillin-resistant *S. aureus*, *Enterococcus faecalis*) and two Gram-negative bacteria (*E. coli* and *K. pneumoniae*). They demonstrated inhibitory activity against all pathogens, with the exception of *E. faecalis*, *K. pneumoniae*, and *E. coli* at a concentration of 5 g/mL. Nevertheless, the outcomes were more favorable than those observed in the control (Chai et al., 2023). Chrysargyris et al. (2016) investigated the toxicity of *L. angustifolia* and *S. fruticosa* EOs from plants grown under different deficit irrigation treatments to a crop pest (two-spotted spider mite, *Tetranychus urticae*) and found that EO from lavender under moderate water deficit caused significantly higher mortality to *T. urticae* than the EO from plants irrigated with adequate water. In contrast, EO from sage showed no significant differences between drought treatments. *T. daenensis* was irrigated at 50% field capacity, and its ability to inhibit two gram-positive (*B. cereus* and *L. monocytogenes*) and two gram-negative (*Proteus vulgaris* and *Salmonella typhimurium*) bacterial strains was evaluated. In general, EO extracted from control plants showed superior antibacterial activity against *B. cereus* and *P. vulgaris* than under drought conditions. On the other hand, thyme EO showed higher activity against *S. typhimurium* and *L. monocytogenes* in stressed than unstressed plants (Pirbalouti et al., 2014). The effect of drought on other biological properties of plant extracts was also investigated. *T. lotocephalus in vitro* cultures grown in culture media supplemented with PEG to simulate drought stress and the antimelanogenic and ultraviolet (UV) protective capacities of the extracts were analyzed. According to the results, all cultures exposed to PEG showed the worst biological activities (Mansinhos et al., 2022a). In addition to these biological properties, MAPs have been increasingly reported as an environmentally friendly strategy for phytoremediation. This is due to the fact that HMs are incorporated into the plant system by chelation or sequestration, neutralizing them. Furthermore, the Lamiaceae is identified as one of the most promising families of MAPs for sustainable phytoremediation. Nevertheless, it is important to note that HMs may infiltrate the food chain and cause dangerous effects. Therefore, it is recommended that non-edible MAPs need to be used in phytoremediation (Mishra and Chandra, 2022).

Although there is some ambiguous information available in the literature regarding the effect of abiotic stress on the biological properties of plants, especially because there are several factors that influence the production of bioactive compounds (e.g., duration/concentration of stress or plant genotype), in most cases, climate-related stress seems to have a positive effect. According to the wide range of drought, salinity, temperature, light, and heavy metal experiments described so far, approximately 70% of the studies showed that these factors positively affected the biological properties of Lamiaceae species, while 30% of these activities were reduced. This evidence could be very favorable to increase the industrial applications of these species. Moreover, this consideration highlights the importance of the production of secondary metabolites as one of the main adaptive mechanisms adopted by plants under stress conditions.

A review of the available data in **Tables 1** **–** **5** indicates that 61% of the studies relate to the investigation of EOs. Furthermore, 1,8-cineole (also known as eucalyptol, one of the main compounds of Lamiaceae EOs) was the most frequently reported compound in Lamiaceae EOs, occurring in 56% of the studies. It was also observed that 1,8-cineole production was stimulated by abiotic stress. This monoterpene, commonly added to cosmetic or perfumery products (e.g., bath additives, mouthwashes, insect repellents), has been shown to have a wide range of pharmacological properties over the last two decades, mainly anti-inflammatory and antioxidant via NF-kB and Nrf2 signaling pathways, respectively. 1,8-Cineole can be used to treat cardiovascular, digestive, and respiratory disorders, as well as Alzheimer’s disease. In clinical trials, this compound has been administered by capsule but has high volatility and poor water solubility, which limits the absorption of 1,8-cineole by oral administration. Thus, the development of suitable formulations for further *in vivo* application is needed (Cai et al., 2021). Linalool is another monoterpene widely used in cosmetics, food additives, and household cleaners (An et al., 2021), which in 59% of the cases increased under abiotic stress (**Tables 1**–**5**). According to An et al. (2021), this terpene can be used as an adjuvant to antibiotics or anticancer treatments due to its low toxicity and protective effects. Linanool has the ability to induce apoptosis in cancer cells while protecting normal cells, and it has also been reported to protect the kidney, liver, and lungs due to its anti-inflammatory activity. Borneol and camphor were the EO components found in the Lamiaceae studies to be more stimulated by environmental factors, particularly drought. In 91% (for borneol) and 85% (for camphor) of the drought and salinity studies reported (**Tables 1**, **2**), the production of this monoterpene increased significantly after exposure to these two environmental stresses. Borneol is widely used in traditional Chinese medicine for its analgesic, antipyretic, and resuscitative effects, as well as in perfumery, medicine, and the chemical industry (Ma et al., 2021). In terms of therapeutic uses, this compound helps to relieve the discomfort associated with hemorrhoids. Furthermore, in 2017, borneol completed the phase III clinical trial of capsules for the prevention and treatment of stable angina (Tasly Pharmaceuticals, Inc, 2017) and more recently, in 2022, the phase III clinical trial of tablets for the treatment of acute ischemic stroke (Peking University Third Hospital, 2022). Camphor is another terpene with multiple applications such as alternative medicine, topical medication, respiratory aerosol, flavoring additive, household cleaner, and perfumery (Chen et al., 2013; McAllister and Burnett, 2015).

With regard to PCs, rosmarinic acid is the most relevant compound in the Lamiaceae plants. According to **Tables 1**–**5**, this phenolic acid was positively affected by drought, salinity, temperature, light, and heavy metals in 81% of the reported studies. Rosmarinic acid has a wide range of applications, from food preservatives to cosmetics, and is also an interesting molecule for pharmaceuticals (Marchev et al., 2021). In terms of its wide range of health-promoting properties, this compound has demonstrated therapeutic benefits in neurodegenerative and liver disorders, cancer, diabetes, and inflammatory diseases (Noor et al., 2022). However, rosmarinic acid has a low lipophilicity, which is not good for its transmembrane penetration and oral availability. In addition, the presence of excipients in cosmetic and pharmaceutical products, as well as the acidic atmosphere of the human digestive tract or many foods, affects the stability of rosmarinic acid, resulting in low bioavailability and a significant decrease in its biological properties (Marchev et al., 2021). Nevertheless, some clinical trials with rosmarinic acid have been conducted in Japan, namely, for cognitive function (JPRN Search Portal, 2016a), dementia (JPRN Search Portal, 2016b), and Alzheimer’s disease (JPRN Search Portal, 2012). Caffeic acid was the second most abundant phenolic compound in Lamiaceae species and was also highly stimulated by abiotic stress (89% of the studies) (**Tables 1**–**5**). Caffeic acid is one of the FDA National Drugs approved and is an active ingredient in at least five products (PubChem, 2023). In 2016, this compound completed phase III clinical trials for tablets as a second-line therapy for immune thrombocytopenia (ITP) (Hou, 2016) and, in 2020, completed phase IV for caffeic acid in combination with high-dose dexamethasone in the treatment of ITP (Hou, 2020). In 2019, it entered phase III as a GASC1 inhibitor for advanced esophageal squamous cell carcinoma (The First Affiliated Hospital of Henan University of Science and Technology, 2020). According to the National Cancer Institute, caffeic acid plays a key role in the development of tumor cells. This phenolic acid is orally bioavailable and has great anti-inflammatory, antioxidant, and antineoplastic properties. When administered, this hydroxycinnamic acid acts as an antioxidant molecule, preventing oxidative stress and consequent DNA damage induced by free radicals (NCI Thesaurus, 2023).

## 6 Conclusions and future prospects

The Lamiaceae MAPs have been used in traditional medicine and perfumery for millennia, and their diverse array of biological properties has led to an increasing interest from various industries. Regrettably, these plants face several environmental stresses, which have a detrimental effect on the quality and quantity of their secondary metabolites. The data presented in this review indicate that the impact of climate-related stresses varies according to several factors, including plant genus, species, genetic background (cultivar), type, level, and duration of stress, as well as experimental conditions. This makes it challenging to generalize about the effect of abiotic stresses on the secondary metabolism of these plants. Nevertheless, the results of the reviewed studies demonstrated that in the majority of cases, these stresses induce the production of secondary metabolites, particularly compounds such as 1,8-cineole, borneol, camphor, linalool, limonene, rosmarinic acid, and caffeic acid. This may potentially enhance the industrial value of the plants. Furthermore, the results indicate the crucial role of these compounds in mitigating the effects of abiotic stress. In the context of climate change scenario, particularly in the Mediterranean basin, the reviewed results are of significant relevance in predicting the response of Lamiaceae plants to environmental changes and assisting the implementation of alternative agricultural and cultivation practices, such as suitable water management, which would allow for the sustainable cultivation of these plants for industrial or other applications. Moreover, several studies have demonstrated that certain Lamiaceae MAPs can accumulate heavy metals without contaminating the EOs. For these reasons, these plants may be considered viable alternatives for phytoremediation while simultaneously serving as a valuable source of bioactive compounds for numerous applications.

## Author contributions

IM: Conceptualization, Writing – original draft. SG: Conceptualization, Funding acquisition, Project administration, Supervision, Writing – review & editing. AR: Conceptualization, Funding acquisition, Project administration, Supervision, Writing – review & editing.
